# Assessment of Chloroquine and Hydroxychloroquine Safety Profiles: A Systematic Review and Meta-Analysis

**DOI:** 10.3389/fphar.2020.562777

**Published:** 2020-10-14

**Authors:** Lu Ren, Wilson Xu, James L. Overton, Shandong Yu, Nipavan Chiamvimonvat, Phung N. Thai

**Affiliations:** ^1^Department of Internal Medicine, Cardiology, University of California, Davis, Davis, CA, United States; ^2^Department of Cardiology, Cardiovascular Center, Beijing Friendship Hospital, Capital Medical University, Beijing, China; ^3^Department of Veteran Affairs, Northern California Health Care System, Mather, CA, United States

**Keywords:** chloroquine, hydroxychloroquine, safety profiles, meta-analysis, adverse events

## Abstract

**Background:**

Chloroquine (CQ) and its derivative hydroxychloroquine (HCQ) have recently emerged as potential antiviral and immunomodulatory options for the treatment of 2019 coronavirus disease (COVID-19). To examine the safety profiles of these medications, we systematically evaluated the adverse events (AEs) of these medications from published randomized controlled trials (RCTs).

**Methods:**

We systematically searched MEDLINE, the Cochrane library, the Cochrane Central Register of Controlled Trials (CENTRAL), and the ClinicalTrials.gov for all the RCTs comparing CQ or HCQ with placebo or other active agents, published before June 20, 2020. The random-effects or fixed-effects models were used to pool the risk estimates relative ratio (RR) with 95% confidence interval (CI) for the outcomes.

**Results:**

The literature search yielded 23 and 19 studies for CQ and HCQ, respectively, that satisfied our inclusion criteria. Of these studies, we performed meta-analysis on 6 studies for CQ and 18 studies for HCQ. We did not limit our analysis to published records involving viral treatment alone; data also included the usage of either CQ or HCQ for the treatment of other diseases. The trials for the CQ consisted of a total of 2,137 participants (n = 1,077 CQ, n = 1,060 placebo), while the trials for HCQ involved 2,675 participants (n = 1,345 HCQ and n = 1,330 control). The overall mild and total AEs were significantly higher in CQ-treated non–COVID-19 patients, HCQ-treated non–COVID-19 patients, and HCQ-treated COVID-19 patients. The AEs were further categorized into four groups and analyses revealed that neurologic, gastrointestinal (GI), dermatologic, and sensory AEs were higher in participants taking CQ compared to placebo, while GI, dermatologic, sensory, and cardiovascular AEs were higher in HCQ-treated COVID-19 patients compared to control patients. Moreover, subgroup analysis suggested higher AEs with respect to dosage and duration in HCQ group. Data were acquired from studies with perceived low risk of bias, so plausible bias is unlikely to seriously affect the main findings of the current study.

**Conclusions:**

Taken together, we found that participants taking either CQ or HCQ exhibited more AEs than participants taking placebo or control. Precautionary measures should be taken when using these drugs to treat COVID-19. The meta-analysis was registered on OSF (https://osf.io/jm3d9).

**Registration:**

The meta-analysis was registered on OSF (https://osf.io/jm3d9).

## Introduction

The 2019 coronavirus disease (COVID-19) is caused by the novel and highly infectious severe acute respiratory syndrome coronavirus 2 (SARS-CoV-2). Since its discovery in December of 2019 in Wuhan, it has now caused a global pandemic. As of June 20, 2020, there were 8,735,394 confirmed cases and 461,786 deaths from the disease, which brings the mortality to approximately 5.3%. Thus, significant efforts have been made to develop a vaccine for SARS-CoV-2. Although it is estimated that vaccine development will take at least 12–18 months (Amanat and Krammer, 2020), two medications—chloroquine (CQ) and hydroxychloroquine (HCQ)—have emerged as possible contenders to treat COVID-19.

Emerging evidence has suggested that these drugs are effective in treating SARS-CoV-2 *in vitro* (Vincent et al., 2005; Liu et al., 2020). Viral replication begins when the virus attaches and penetrates the host cell. In the case of SARS-CoV-2, it uses its surface unit (S1) of the S protein to attach to the angiotensin-converting enzyme 2 (ACE2) receptor, which facilitates viral entry (Hoffmann et al., 2020). When African green monkey kidney VeroE6 cells were pretreated for an hour with CQ or HCQ prior to four different multiplicities of infection by SARS-CoV-2, both drugs prevented viral entry as well as post-entry stages of SARS-CoV-2 infection (Liu et al., 2020). Inhibition of viral entry may be due to the interference of terminal glycosylation of the ACE2 receptor (Vincent et al., 2005). Additionally, CQ and HCQ can alkalinize the phagolysosome, which disrupts the pH-dependent steps of viral fusion and uncoating—processes that are absolutely essential for viral replication (Rolain et al., 2007).

Moreover, both CQ and HCQ have immunomodulatory properties (Schrezenmeier and Dörner, 2020) that may be beneficial in extreme, life-threatening COVID-19 cases. Indeed, there has been a recent surge in COVID-19 patients with severe hyper immune activity, known as the *cytokine storm syndrome* (Mehta et al., 2020). In this patient population, immunosuppression is likely to be beneficial, since the over-active immune response is paradoxically causing more harm than benefit to the patients. Therefore, CQ and HCQ have recently become appealing due to their antiviral and anti-inflammatory properties, which may help treat COVID-19, especially under dire circumstances.

Although the promising findings suggest that CQ and HCQ are great candidates, much concern exists regarding their mechanisms, effective dosing regimen, clinical efficacy, and adverse effects with respect to COVID-19. Indeed, our current knowledge on CQ and HCQ are derived from non–COVID-19 patients treated for diseases such as malaria, rheumatoid arthritis, and systemic lupus erythematosus. The rise in popularity of these drugs as potential medications to treat COVID-19 and the current desperate need for better therapeutics have fueled rapid and ongoing research and clinical trials (Cortegiani et al., 2020) to further elucidate their antiviral and anti-inflammatory properties, pharmacodynamics, and safety profiles with respect to COVID-19.

Currently, the safety profiles of these drugs for COVID-19 are not entirely known due to the lack of large clinical trials, as well as sparse randomized controlled trials (RCTs). Moreover, the drugs have a narrow therapeutic range, which presents another challenge when using these drugs (Frisk-Holmberg et al., 1983; Touret and de Lamballerie, 2020). We therefore designed a meta-analysis to assess CQ/HCQ AEs in non–COVID-19 and COVID-19 patients. We believe that despite the shortcomings, comprehensively evaluating the existing data on these drugs can provide powerful and valuable insights regarding their safety profiles, which will not only drive future clinical trials, but also help health professionals make informed decisions.

## Methods

The meta-analysis was conducted in accordance with the Preferred Reporting Items for Systematic Reviews and Meta-Analyses (PRISMA) guidelines. The PRISMA flow diagram was included in the **Supplementary Materials**.

## Literature Search and Inclusion Criteria

A comprehensive search strategy was designed to retrieve relevant clinical data from published literature. Our objective was to identify all RCTs that compared the safety profiles of CQ or HCQ with placebo or other active agents. We searched MEDLINE, the Cochrane Library, the Cochrane Central Register of Controlled Trials (CENTRAL), and the ClinicalTrials.gov for all the RCTs comparing CQ or HCQ with placebo or other active agents, published before June 20, 2020. We also searched conferenced proceedings to acquire relevant papers. Medical subject headings (MeSH terms) and keywords such as “randomized controlled trial,” “adverse effects,” “tolerability,” “toxicity,” and “side effects” were used. This review was not restricted to studies conducted in the English language; it includes records from any countries that compared CQ or HCQ with placebo or other active agents, since there is a wealth of information in RCTs from many different countries.

Due to the lack of large clinical trials and small numbers of RCTs, we decided to include all the RCTs reporting adverse events (AEs) in patients with different disease conditions, including rheumatoid arthritis, systemic lupus erythematosus, infectious diseases such as HIV infection, and immune diseases such as Primary Sjögren’s Syndrome. We included all RCTs in adult patients that compared CQ or HCQ with other active agents or placebo.

To be included in the analysis, the study had to fulfill the following criteria: (1) randomized trials which could be open-label, single-blind, double-blind, or parallel group studies; (2) use of CQ or HCQ as one of the interventions; (3) studies comparing CQ or HCQ with placebo or other active agents; and (4) available data on safety and tolerability data for CQ or HCQ.

Studies were excluded from meta-analysis if: (1) they presented data on children only; (2) they lacked placebo group; (3) study did not present safety and tolerability outcomes; (4) full text could not be sourced; (5) CQ or HCQ was used in combination with other drugs.

## Data Collection and Outcome Measures

Bibliographic details and abstracts of all citations retrieved by the literature search were downloaded to EndNote X9. All studies were screened and evaluated by two independent reviewers (LR and PT), which were then checked by a third reviewer (SY). Discrepancies were resolved by discussion in group conferences. Completed data were then thoroughly checked by two additional reviewers (WX and JO). Data including first author, year of publication, trial design, country where studies took place, purpose of treatment, trial duration, dosage regimen, outcomes and AEs were extracted using a standardized form and presented in table format. Safety evaluation included monitoring of AEs and vital signs. Withdrawals due to AEs were reported.

## Study Quality Assessment and Risk of Bias

Risk of bias in the individual studies included for meta-analysis was assessed using the Cochrane risk assessment tool (Higgins et al., 2011). The assessment was performed by two independent reviewers (WX and JO) and further checked by two additional reviewers (LR and PT). The completed information is provided in **Supplementary Table S1**.

## Statistical Analysis

Comparison of safety and tolerability outcomes was made between interventions by pooling data from studies using a direct meta-analysis technique. All terminology used when analyzing data was in accordance with the Common Terminology of Clinical Adverse Events handbook. Outcomes were summarized as relative risk ratios. Random-effects model (Barili et al., 2018) was used to pool the risk estimates relative ratio (RR) with 95% confidence interval (CI) for the outcomes. If I^2^ ≥ 40%, the heterogeneity is high. Although we did not alter this in our software output, but I^2^ < 0% may be considered as I^2^ = 0%. We analyzed results from RCTs that had placebo controls. Subgroup analyses were performed to see the effects of different age, duration, and dosage on relative risk of total AEs. For the HCQ studies, subgroup analysis of different pathologies on relative risk on total AEs was also assessed. Random-effects meta-regression models were used to test whether the relative risk of total AEs was affected by the age, dosage, or trial duration. Comparisons with no events in either group were excluded. I^2^ statistics was included in all the meta-analyses that were performed, which is a percentage of variance attributed to study heterogeneity. Heterogeneity tests were performed. Publication bias was conducted with restricted maximum likelihood method. Sensitivity analyses were conducted by leaving one study out, or by removing all studies with zero events. Analyses were performed using STATA 16 (Stata, College Station, TX, USA). Sensitivity analyses was performed with OpenMeta[Analyst] (CEBM, Brown University) or STATA 16.

## Results

### Process of Identifying Eligible Clinical Trials

**Figure 1:**
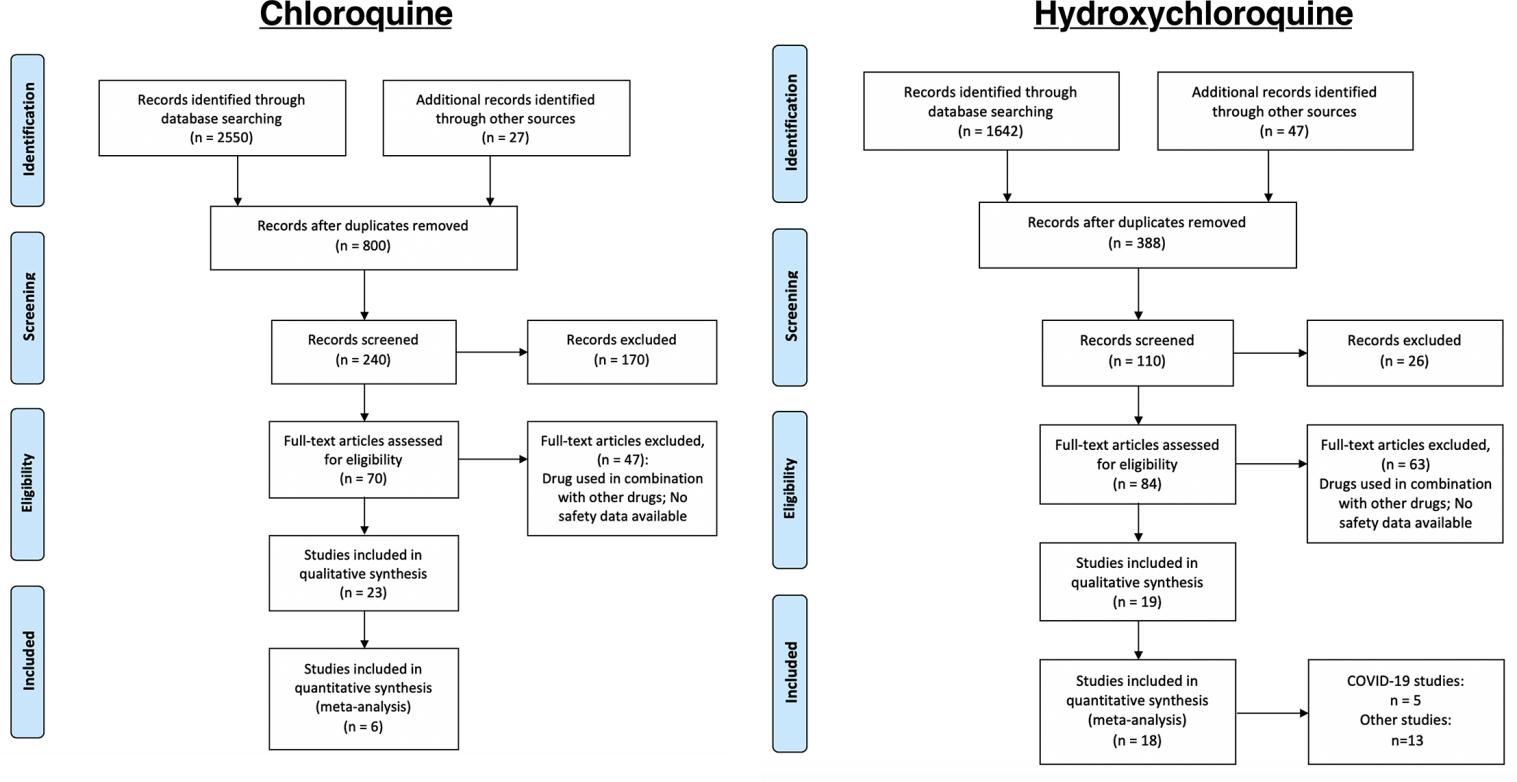
Process of identifying eligible clinical trials. Records were identified through MEDLINE, CENTRAL, and ClinicalTrials.gov. We used the same process of study collection for both CQ and HCQ. We performed an initial screening, followed by a more stringent screening using our selection criteria. The studies that remained after all the exclusion were the ones used for data extraction. In total, we identified 23 and 17 studies for CQ and HCQ, respectively, which are described in, **Table 1** and **2**. Of those studies, 6 CQ and 16 HCQ records are controlled RCTs, so we used these studies for our data analysis.

We identified records that involved either CQ (n = 2,577) or HCQ (n = 1,689). Of the published records we identified, we initially screened them through the titles and abstracts to examine if they were relevant to our objective of identifying safety profiles for CQ and HCQ. Therefore, 170 and 26 records were initially excluded for CQ and HCQ, respectively. Of the remaining ones (n = 70 for CQ and n = 84 for HCQ), we performed a more thorough review using the inclusion and exclusion criteria described in the methods. In total, 23 CQ and 19 HCQ studies satisfied our requirements. The literature search strategy used for each database was listed in the supplementary materials. Therefore, a total of 6 studies and 18 studies were used for data extraction for CQ and HCQ, respectively (**Figure 1**).

### Characteristics of Trials, Patients, and Interventions

**Table 1** describes the characteristics of the trials, patients, and interventions of CQ, while **Table 2** describes the same parameters for HCQ. The trials indicated with asterisks next to the primary author’s last name were the trials used for our meta-analyses. As shown in the tables, we did not restrict our systematic review to just the United States. Additionally, investigators used CQ as treatment options for breast cancer (Amanat and Krammer, 2020), malaria (Beck et al., 2020), hepatitis (Vincent et al., 2005), viral infections (Rolain et al., 2007), and lupus erythematosus (Amanat and Krammer, 2020). To conduct our meta-analysis for CQ, we used 6 double-blinded, placebo-controlled, randomized studies that used CQ for the treatment of breast cancer, autoimmune hepatitis, dengue fever, and influenza. Age of participants ranged from 22 to 57 years old. Dosing regimen ranged from approximately 107 mg/day to 1,000 mg/day. Of these studies, general findings reported in the studies noted that CQ did not have a significant effect when compared with placebo. However, of the studies that compared CQ with other medications, the authors noted that CQ was generally more effective.

**Table 1:** Characteristics of CQ studies.

Study	Study Type	Country	Treated Disorder (n patients)	Trial Duration (weeks)	Dosage	Summary of Outcomes	Intervention (n of patients)	Age (mean or median)	Total n of AEs	Total n of serious AEs
*Arnaout et al. (2019**)**	Double-Blinded, Placebo-Controlled, Randomized, Window of Opportunity Trial	Canada	Breast Cancer (70)	2–6	500 mg/day CQ or Placebo for 2–6 weeks	No significant effects	CQ: 46	57.4 ± 9.7	35	0
							Control: 24	55.7 ± 8.4	8	0
Divala et al. (2018**)**	Open-Label, Randomized, Single-Centered, Three-Armed	United States/Malawi	Placental Malaria (900)	20–28 of gestation to birth	Days 1-2: 600 mg Day 3: 300 mg≥ 4 weeks later (CQ-IPTt) or 600 mg at enrollment, then 300 mg/week until delivery (prophylaxis)	CQ IPTp was not better than SP-IPTp	CQ: 600	33.00 ± 12.11	5	0
							SP-IPTp: 300	33.95 ± 11.91	3	0
*Terrabuio et al. (2019**)**	Double-Blinded, Interventional, Parallel-Group, Placebo-Controlled, Randomized, Single-Centered	Brazil	Autoimmune Hepatitis (AIH) (61)	156.4	250 mg/day for 36 months	CQ safely reduced relapse risk of AIH; no subgroup with greater benefit from CQ use	CQ: 31	37.7 ± 16.1	17	0
							Control: 30	39.1 ± 16.9	5	0
Abreha et al. (2017**)**	Randomized	United States/Ethiopia	Vivax Malaria (398)	6	25 mg/kg over 3 days	Primaquine (PQ) + CQ or Artemether-Lumefantrine (AL) reduced vivax malaria recurrence 5 folds over 1 year	CQ: 206	Median: 18	165	0
							AL or AL+PQ: 192	CQ+PQ: 17 AL: 18 AL+PQ: 18	165	0
Grigg et al. (2018**)**	Open-Label, Randomized, Two-Armed	Australia/Malaysia	Uncomplicated *Plasmodium Knowlesi* Malaria (123)	6	25 mg/kg at enrollment, 6, 24, and 48 h	Artemether-Lumefantrine (AL) was effective at treating *knowlesi* malaria	CQ: 58	Median: 31	25	0
							AL: 65	Median: 30	29	0
Valecha et al. (2016**)**	Multicentric, Open-Label, Phase III Study	India	Acute, Uncomplicated *Plasmodium Vivax* Malaria (317)	≥6	CQ: 4 doses (total 10 tablets of 250 mg each) for 3 days	FDC of arterolane maleate (AM) and PQP cures *vivax* marlaria	CQ: 158	33.7 ± 13.45	135	0
							AM+PQP: 137	33.2 ± 11.81	127	4
Siqueira et al. (2017**)**	Open-Label, Non-Inferiority, Randomized	Brazil	*Vivax* Malaria (380)	6	25 mg/kg over 3 days	Artesunate-Amodiaquine (ASAQ) is more effective than CQ at preventing *P. vivax* infection	CQ: 189	34.7 ± 15.9	52	0
							ASAQ: 190	35.7 ± 16.4	68	5
Peymani et al. (2016**)**	Triple-Blinded, Placebo-Controlled, Randomized, Pilot	Iran	Hepatitis C (10)	8	150 mg/day for 8 weeks	CQ was potentially safe for HCV non-responders	CQ: 6	49	0	7
							Control: 13	50	0	0
Grigg et al. (2016**)**	Open-Label, Randomized	Australia/Malaysia	Uncomplicated *Plasmodium Knowlesi* Malaria (252)	6	25 mg/kg at enrollment, 6, 24, and 48 h after treatment	Artesunate-Mefloquine (AM) was highly effective at treating *P. Knowlesi* Malaria	CQ: 125	Median: 32	316	0
							AM: 127	Median: 33	302	2
Chopra et al. (2014**)**	Assessor-Blinded, Parallel Efficacy, Randomized, Two-Armed	India	Musculoskeletal Pain and Arthritis Following *Chikungunya* virus infection (70)	24	250 mg/day for 24 weeks	No significant improvement over meloxicam	CQ: 38	50.2	7	0
							Meloxicam: 32	45.4	5	0
*Borges et al. (2013**)**	Double-Blinded, Placebo-Controlled, Randomized	Brazil	Dengue (129)	3 days	1,000 mg/day for 3 days	CQ reduced pain; improved well-being of patients; but did not affect disease duration	CQ: 63	31.64 ± 11.74	2	0
							Control: 66		0	0
*Paton et al. (2011**)**	Double-Blinded, Placebo-Controlled, Randomized	Singapore	Influenza (1,516)	12	Week 1: 500 mg/day Weeks 2–12: 500 mg/week	No significant effects	CQ: 757	23.6	341	3
							Control: 759	23.5	249	5
Awab et al. (2010**)**	Open-Label, Perspective, Randomized	Afghanistan	*Vivax* Malaria (536)	8	25 mg/kg for 3 days	CQ was effective for *Vivax* Malaria treatment	CQ: 268	Mean: 11	15	0
							DP: 268	Median: 12	2	0
*Tricou et al. (2010**)**	Double-Blinded, Placebo-Controlled, Randomized	Vietnam	Dengue (307)	3 days	Days 1–2: 600 mg Day 3: 300 mg	CQ did not reduce viraemia/NSI antigenaemia (AG) in dengue patients	CQ: 153	22	18	0
							Control: 154	22	6	0
*De Lamballerie et al. (2008**)**	Double-Blinded, Placebo-Controlled, Randomized	France	Chikungunya Infection (54)	5 days	Days 1–3: 600 mg/day Days 4-5: 300 mg/day	No significant effect on acute Chikungunya infection	CQ: 27	Range: 18-65	7	0
							Control: 27		0	0
Villegas et al. (2007**)**	Double-Blinded, Placebo-Controlled, Randomized	Thailand	*Vivax* Malaria in Pregnancy (1,000)	Weekly till delivery	500 mg/week	CQ was safe and effective as a prophylaxis against *P. Vivax* during pregnancy	CQ: 500	26.1 ± 6.4	2	0
							Control: 500	25.4 ± 6.3	1	0
Laufer et al. (2006**)**	Randomized	United States/Malawi	Uncomplicated *Plasmodium Falciparum* Malaria (210)	4	Days 0–1: 10 mg/kg Day 2: 5 mg/kg	CQ was effective in Malawi after 12 years	CQ: 80	2.6 ± 2.2	0	0
							Sulfadoxine-Pyrimethamine: 87	2.9 ± 2.2	0	0
Dunne et al. (2005**)**	Double-Blinded, Randomized	India	*Plasmodium Vivax* Malaria (199)	4	Days 1–2: 600 mg Day 3: 300 mg	CQ was tolerated as well, but was more effective	CQ: 102	30.0 ± 11.8	33	2
							Azithromycin: 97	31.7 ± 11.6	20	0
Mucenic et al. (2005)	Pilot Study	Brazil	Remission of Autoimmune Hepatitis (32)	≥52	250 mg/day for ≥12 months	CQ group had lower relapse frequency	CQ: 14	27.29 ± 15.23	18	0
							Control: 18	26 ± 13.59	0	0
Bezerra et al. (2005**)**	Double-Blinded, Randomized	Brazil	Lupus Erythematosus (33)	26.1	250 mg/day for 6 months	Clofazimine (CFZ) equally as effective as CQ diphosphate (CDP)	CQ: 17	34.4	21	0
							CFZ: 16	34	21	0
Llanos-Cuentas et al. (2001**)**	Open-Label, Randomized, Comparison	Peru	Acute *Plasmodium Falciparum* Malaria (29)	4	Day 1: 600 mg Days 2–3: 300 mg	Atovaquone/Proguanil (A/P) much more effective than CQ	CQ: 14	Range: 12–65	29	0
							A/P: 15		26	1
Hatz et al. (1998**)**	Comparative, Open, Parallel Group, Randomized, Single-Centered	Switzerland/Tanzania	Acute *Plasmodium Falciparum* Malaria (26)	4	Day 1: 10 mg/kg Days 2–4: 5 mg/kg	CGP-56697 highly effective against *P. Falciparum* in this part of Tanzania	CQ: 130	Median: 2	17	0
							CGP-56697: 130	Median: 2	6	0
Kofi Ekue et al. (1983**)**	Double-Blinded, Randomized	Zambia	Symptomatic *Falciparum* Malaria (99)	6	Day 1: 900 mg Days 2–3: 300 mg	No significant differences between MQ and CQ	CQ: 49	Range: 13-51	62	0
							MQ: 50		45	0

**Table 2:** Characteristics of HCQ studies.

Study	Study Type	Country	Treated Disorder (n patients)	Trial Duration (weeks)	Dosage	Summary of Outcomes	Intervention (n of patients)	Age	Total n of AEs	Total n of serious AEs
*Boulware et al. (2020**)**	Randomized, double-blind, placebo-controlled trial	United States and Canada	COVID-19	1	800 mg once, then 600 mg 6 to 8 h later, then 600 mg daily	HCQ did not prevent illness compatible with COVID-19	HCQ: 349	41	140	0
							Control: 351	40	59	0
*Jun et al. (2020**)**	Randomized Pilot Study	China	COVID-19 (30)	1	400 mg/day for 5 days	Prognosis of common COVID-19 patients is good	HCQ: 15	50.5 ± 3.8	4	0
							Control: 15	46.7 ± 3.6	3	0
*Cavalcanti et al. (2020**)**	Multicenter, randomized, open-label, controlled trial	Brazil	COVID-19	1	400 mg twice daily for 7 days	HCQ did not improve clinical status compared with standard care	HCQ: 221	51.3 ± 14.5	67	2
							Control: 227	49.9 ± 15.1	40	2
*Mitjà et al. (2020**)**	Multicenter, open label, randomized controlled trial	Spain	COVID-19	1	800 mg on day 1, 400mg daily for 6 days	No benefit was observed with HCQ beyond the usual care	HCQ: 169	41.6	121	8
							Control: 184	41.7	16	12
*Tang et al. (2020**)**	Multicenter, open label, randomized controlled trial	China	COVID-19	2-3	1,200 mg/d for 3 days and then 800 mg/d	HCQ did not result in a significantly higher probability of negative conversion of virus than control	HCQ: 70	48.0	21	2
							Control: 80	44.1	7	0
*Boonpiyathad et al. (2017**)**	Single-Blind, Placebo-Controlled, Randomized	Thailand	Anti-Histamine Refractory Chronic Spontaneous Urticaria (CSU) (55)	12	400 mg/day for 12 weeks	HCQ was effective as an adjunct treatment for CSU	HCQ: 46	33.00 ± 12.11	5	0
							Control: 24	33.95 ± 11.91	3	0
*Wasko et al. (2015**)**	Double-Blinded, Parallel-Arm, Placebo-Controlled, Randomized	United States	Pre-Diabetes (32)	13 ± 1	400 mg/day for 13 ± 1 weeks	HCQ improved both ß-cell function and insulin sensitivity in non-diabetic patients	HCQ: 17	>18	3	0
							Control: 15		3	0
*Gottenberg et al. (2014**)**	Double-Blinded, Parallel-Group, Placebo-Controlled	France	Primary Sjogren’s Syndrome (120)	48	400 mg/day Placebo or HCQ for 24 weeks, then 400 mg/day HCQ for 24 weeks	No significant effects	HCQ: 56	56.3 ± 11.9	5	5
							Control: 64	55.6 ± 13.9	7	7
*Solomon et al. (2014**)**	Blinded, Crossover, Randomized	United States	Rheumatoid Arthritis and Insulin Resistance (30)	16	6.5 mg/kg HCQ or placebo daily for 8 weeks, then crossover to other arm for 8 weeks	No significant change in insulin resistance; minor improvements to total LDL cholesterol	15 (HCQ → Placebo)	56 ± 11.4	2	0
							15 (Placebo → HCQ)	56 ± 11.4	0	0
*Rotaru et al. (2014**)**	Randomized, Pilot, Triple Masking	United States	Kidney Failure, Chronic Cardiovascular Disease Arteriosclerosis (8)	25	200 mg/day for 10 days ± 4 days, then 200 mg twice daily for 6 months	Terminated (Lack of Funding)	HCQ: 7	18-65: 4 >65: 3	2	0
							Control: 1	18–65: 1	0	0
*Paton et al. (2012**)**	Double-Blinded, Randomized, Placebo-Controlled	United Kingdom	HIV (83)	48	400 mg/day for 48 weeks	No significant effects	HCQ: 42	37.1 ± 7.7	41	0
							Control: 41	38.3 ± 10.8	26	0
*Fong et al. (2007**)**	Double-Blinded, Placebo-Controlled, Randomized	United States	Chronic Graft-Versus-Host Disease (95)	55	121 days at 800 mg/day	No effects	HCQ: 46	48	1	0
							Control: 49	46	1	0
*Gerstein et al. (2002**)**	Double-Blinded, Placebo-Controlled, Randomized	Canada	Type 2 Diabetes Mellitus (135)	78.2	300 mg first month, 450 mg s, and 600 mg third, daily	HCQ improved glycemic control in patients with poorly controlled type 2 diabetes	HCQ: 69	57.5	3	0
							Control: 66	57.5	1	0
*Van Gool et al. (2001**)**	Double-Blinded, Parallel-Group, Multicenter	The Netherlands	Dementia in Early Alzheimer’s Disease (168)	78.2	<65 kg: 200 mg/day >65 kg: 400 mg/day; 18 months	No significant effects	HCQ: 83	70.4 ± 8.3	20	5
							Control: 85	70.7 ± 8.5	15	2
*Sperber et al. (1995**)**	Double-Blinded, Placebo-Controlled, Randomized	United States	HIV-1 (40)	8	800 mg/day for 8 weeks	HIV-1 RNA declined significantly in the HCQ group over 8 weeks; increased in placebo group	HCQ: 19	39.1 ± 6.6	0	0
							Control: 19	40.6 ± 12.5	0	0
*The HERA Study Group (1995**)**	Double-Blinded, Placebo-Controlled, Randomized	Canada	Early Rheumatoid Arthritis (120)	36	200 mg/day for 2 weeks. If no side effects, 400 mg/day	Improved pain and disability of recent arthritis	HCQ: 59	53 ± 13.5	25	1
							Control: 60	53 ± 14.8	19	0
*Clark et al. (1993**)**	Double-Blinded, Placebo-Controlled, Randomized	Mexico	Early Rheumatoid Arthritis (126)	24	400 mg/day for 24 weeks	HCQ effectively improved early rheumatoid arthritis	HCQ: 65	39	28	0
							Control: 65	36	28	1
*Kruize et al. (1993**)**	Double-Blinded, Crossover, Placebo-Controlled	The Netherlands	Primary Sjogren’s Syndrome (19)	52.2	400 mg/day for 12 months	No significant effects	10 (HCQ → Placebo)	52.8 ± 16.1	0	1
							9 (Placebo → HCQ)	51 ± 15.8	0	0
Faarvang et al. (1993**)**	Double-Blinded, Multicenter, Parallel-Group, Placebo-Controlled, Randomized	Denmark	Rheumatoid Arthritis (91)	26.1	250 mg/day HCQ and 2g/day Placebo OR 250 mg/day + S for 6 months	HCQ and Sulphasalazine (S) had no improvement over HCQ alone	62 (HCQ + Placebo & HCQ + Sulphasalazine)	61	7	0
							29 (Placebo + Sulphasalazine)	61	0	0

Similarly, the 19 HCQ studies (**Table 2**) that we examined were conducted from a plethora of countries and used HCQ to treat a myriad of disorders, which included dermatologic disorders (Amanat and Krammer, 2020), rheumatoid arthritis (Rolain et al., 2007), HIV (Liu et al., 2020), Primary Sjögren’s Syndrome (Liu et al., 2020), graft-versus host disease (Amanat and Krammer, 2020), diabetes (Liu et al., 2020), chronic spontaneous urticaria (Amanat and Krammer, 2020), dementia (Amanat and Krammer, 2020), kidney failure (Amanat and Krammer, 2020), cardiovascular disease (Amanat and Krammer, 2020), and COVID-19 (Rolain et al., 2007). To conduct our meta-analysis for HCQ, we used RCTs that were pilot studies (one specifically for COVID-19), 3 open-label, 1 single-blinded, and the rest double-blinded. These studies are shown with asterisks next to the primary author’s last name in the table. For these particular records, age of participants ranged from 33 to 70 years. Dosage schedule ranged from 200 mg/day to 1,200 mg/day, with a mode of 400 mg/day, depending on the treated disorder. COVID-19 patients required a higher dosage (>400 mg/day), but a lower duration (<2 weeks) relative to other treated conditions. General outcomes from about a third of the studies revealed that HCQ had no significant effect, while the rest of the studies showed that it was effective for the disorders.

### Mild, Severe, Total AEs, and Withdrawals Due to AEs From Trials Involving CQ and HCQ in Non–COVID-19 Patients

**Figure 2:**
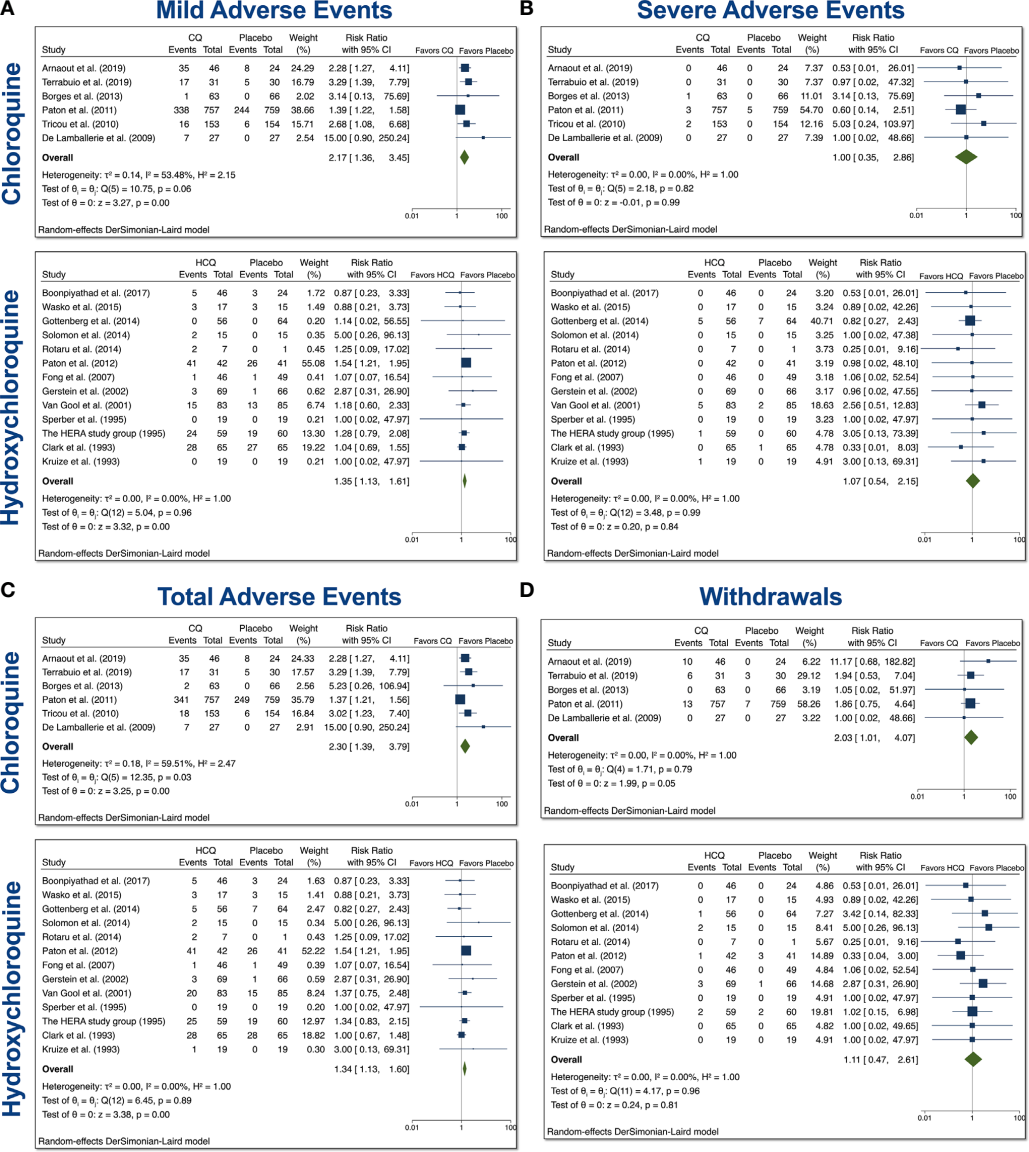
Mild, severe, total AEs, and withdrawals due to AE from trials involving CQ and HCQ in non–COVID-19 patients. We performed 6 comparisons between CQ and placebo and 16 comparisons between HCQ and placebo, as evident in the forest plots. AEs were divided into **(A)** mild, **(B)** severe, and **(C)** total. **(D)** Additionally, we also examined withdrawals from trials due to AEs. Meta-analyses were performed. We tested heterogeneity between trials, as well as overall effect. Statistical data are displayed in the forest plots.

The CQ meta-analyses of mild, serious, total AEs, and withdrawals due to AEs were based on 6 comparisons between CQ and placebo (control), while the HCQ meta-analyses of mild, serious, total AEs, and withdrawals due to AEs were based on 16 comparisons between HCQ and placebo (control), as depicted in **Figure 2**. When assessing mild AE (**Figure 2A**), the overall relative risk (RR) of CQ compared with placebo was 2.17 (95% CI 1.36–3.45, p < 0.01), while the overall RR of HCQ compared with placebo was 1.35 (95% CI 1.13–1.61, p < 0.01). The RR for severe AEs (**Figure 2B**), however, was insignificant for both drug usage when compared with placebo. When assessing total AEs of either drug compared with placebo (**Figure 2C**), the combined RR for CQ was 2.30 (95% CI 1.39–3.79, p < 0.01), while for HCQ it was 1.34 (95% CI 1.13–1.60, p < 0.01). There was statistical evidence of overall heterogeneity between CQ trials with regard to total AEs (I^2^ = 59.51%). Withdrawals due to AEs was near significant with CQ compared with placebo. As evident in **Figure 2D**, the overall RR was 2.03 (95% CI 1.01–4.07, p = 0.05). There was no evidence of heterogeneity (I^2^ = 0%). Taken together, these data suggest that both drugs induced higher mild and total AEs as compared to control.

### System Analyses From Trials With CQ and HCQ in Non–COVID-19 Patients

**Figure 3:**
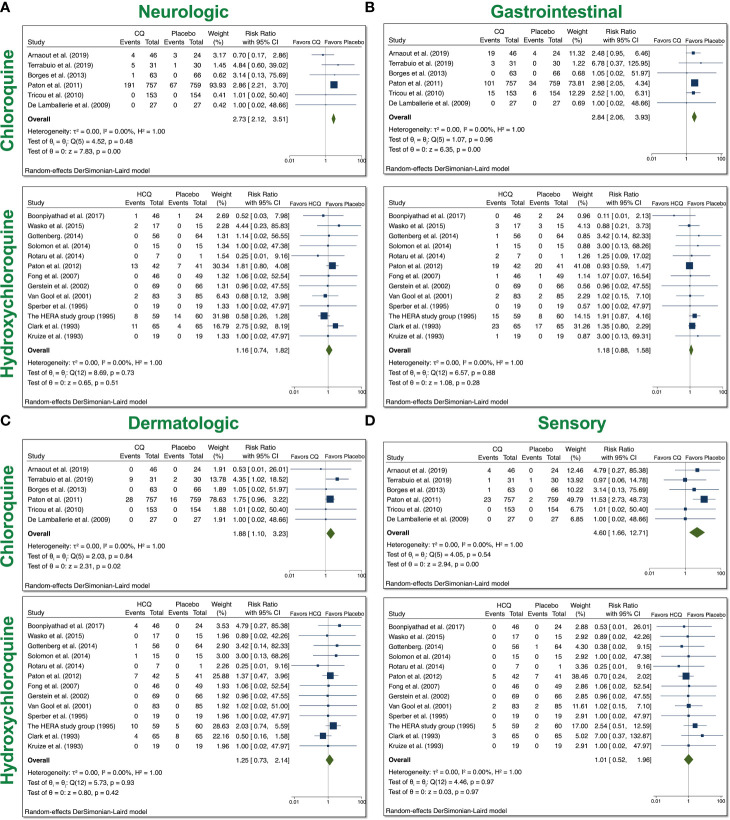
System analyses from trials with CQ and HCQ in non–COVID-19 patients. We performed 6 comparisons between CQ and placebo and 16 comparisons between HCQ and placebo, as evident in the forest plots.. AEs were divided into four groups: **(A)** neurologic, **(B)** gastrointestinal (GI), **(C)** dermatologic, and **(D)** sensory AEs. Using meta-analyses, we tested heterogeneity between trials, as well as overall effect. Statistical data are displayed in the forest plots.

Based on the reported AEs, we divided our analyses to examine four groups: neurologic, gastrointestinal (GI), dermatologic, and ophthalmic AEs. Neurologic AEs reported by participants included headache, dizziness, neuropathy/seizure, or other central nervous system (CNS) related AEs; GI AEs included vomiting, nausea, abdominal pain, diarrhea, liver dysfunction, or non-specific GI AEs; dermatologic AEs included rash, itchiness, dryness; and sensory AEs included blurred vision, pain, or auditory problems. With the usage of CQ, there was a significant increase in all four groups of AEs (**Figure 3**). The overall RR was 2.73 (95% CI 2.12–3.51, p < 0.01) for neurologic AEs; 2.84 (95% CI 2.06–3.93, p < 0.01) for GI AEs; 1.88 (95% CI 1.10–3.23, p < 0.05) for dermatologic AEs; and 4.60 (95% CI 1.66–12.71, p < 0.01) for sensory AEs. No heterogeneity between the trials were observed. With the usage of HCQ, there was no significant increase in any of the groups that we examined. These data suggest that patients treated with CQ experienced more neurologic, dermatologic, ophthalmic, and GI AEs relative to placebo control, while patients treated with HCQ did not experience more of these AEs compared to control.

Further analyses on heterogeneity, as well as publication bias, can be seen in **Supplementary Figures S4–S7**. Study and quality assessment can be seen in **Supplementary Table S1**. Risk of bias was assessed using eight different categories with judgment of risk indicated as either positive (low risk) or negative (high risk). The majority of the studies used in this meta-analysis were deemed low risk by two independent reviewers. We therefore believe that plausible bias would unlikely affect the key findings of the current study.

### Mild, Severe, Total AEs, and Withdrawals Due to AEs From COVID-19 Studies Involving HCQ

**Figure 4:**
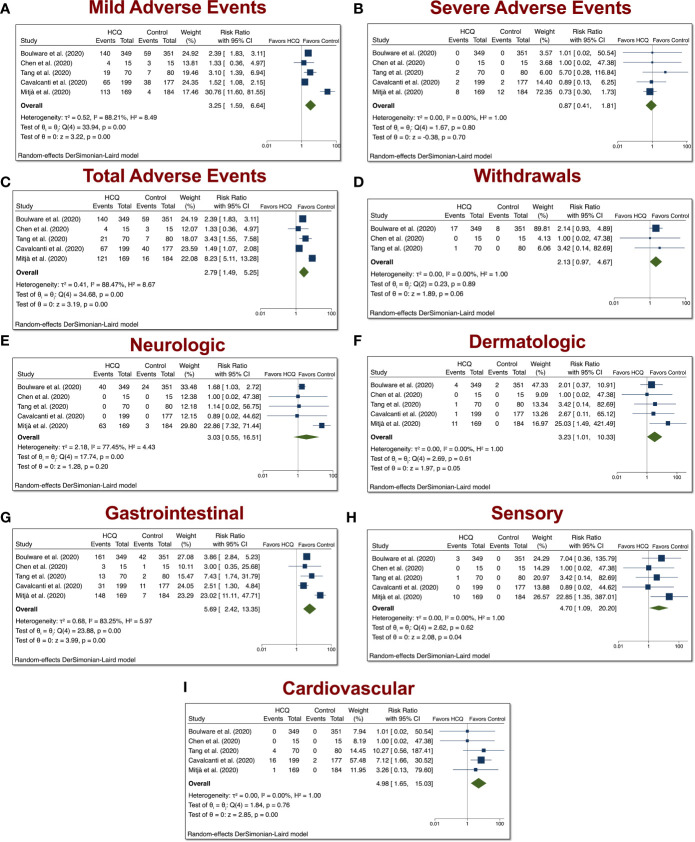
Mild, severe, total AEs, and withdrawals due to AEs from COVID-19 studies involving HCQ. The HCQ meta-analyses of **(A)** mild, **(B)** severe, **(C)** total, **(D)** withdrawals due to AEs, **(E)** neurologic, **(F)** dermatologic, **(G)** gastrointestinal, **(H)** sensory, **(I)** and cardiovascular AEs were based on five comparisons between HCQ and control in COVID-19 studies.

The HCQ meta-analyses of mild, serious, total AEs, and withdrawals due to AEs were based on five comparisons between HCQ and placebo (control) in COVID-19 studies, as depicted in **Figure 2**. When assessing mild AE (**Figure 4A**), the overall relative risk (RR) of HCQ compared with placebo was 3.25 (95% CI 1.59–6.64, p < 0.01). The RR for severe AEs (**Figure 4B**), however, was insignificant. When assessing total AEs of HCQ compared with placebo (**Figure 4C**), the combined RR was 2.79 (95% CI 1.49–5.25, p < 0.01). Withdrawals due to AEs was not significant. As in **Figure 4D**, the overall RR was 2.13 (95% CI 0.97–4.67, p = 0.06). There was no evidence of heterogeneity (I^2^ = 0%). Taken together, these data suggest that HCQ induced higher mild and total AEs as compared to control in patients with COVID-19.

Stratification of the AEs into distinct groups revealed that COVID-19 patients treated with HCQ exhibited increased dermatologic (overall RR 3.23, 95% CI 1.01–10.33, p < 0.01), GI (overall RR 5.69, 95% CI 2.42–13.35, p < 0.01), sensory (overall RR 4.70, 95% CI 1.09–20.20, p < 0.01), and cardiovascular (overall RR 4.98, 95% CI 1.65–15.03, p < 0.01) AEs relative to control patients. There was evidence of heterogeneity between trials with respect to GI AEs (I^2^ = 84.57%).

### Stratification of All AEs

**Figure 5:**
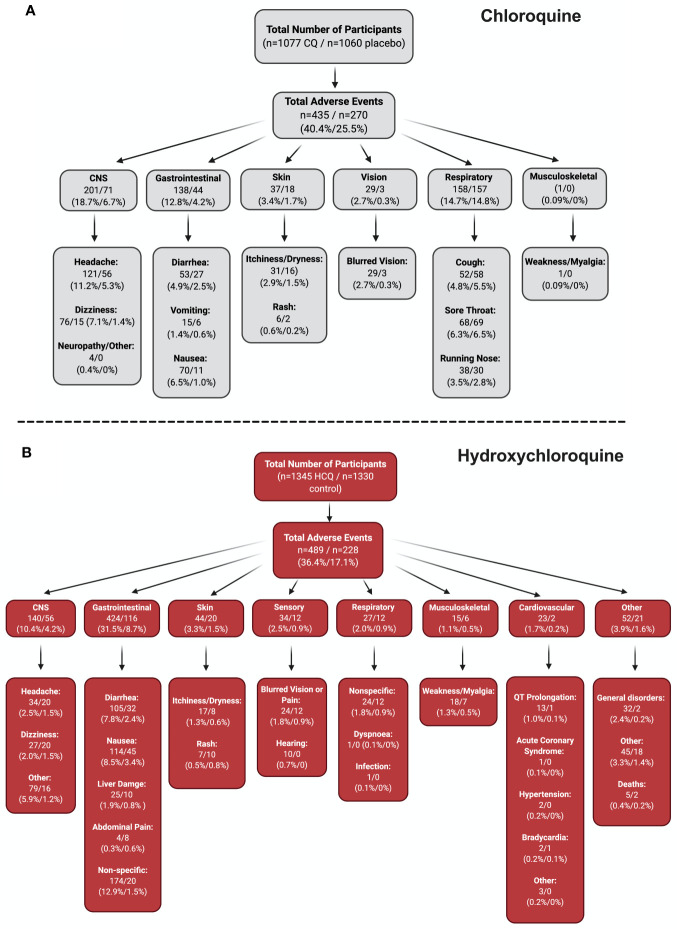
Stratification of all AE. To fully appreciate the wealth of data regarding CQ and HCQ AE, we divided the AE into different categories. Panel **(A)** depicts the data for CQ, while panel **(B)** shows the data for HCQ. Both panels begin with the total number of participants in the studies (n = 6 CQ, n = 18 HCQ), which is then followed by the total number of AE. The AE were then divided into different systems, which is then broken down into specific AE. Figure was generated using BioRender.

To fully appreciate the wealth of information from the RCTs from all the CQ/HCQ reports, we constructed a flow chart that contains information on the number of participants who experienced a certain AE, as well as the percentages. Four groups (CNS, GI, skin, and sensory) underwent meta-analyses (**Figure 4**), since they had robust records in the studies that we examined. In **Figure 5**, panels A and B show the charts for CQ and HCQ, respectively. The 6 CQ studies contained a total of 1,077 participants for CQ-treated group and it contained a total of 1,060 participants for placebo-treated. Of these participants, 435 (40.4%) and 270 (25.5%) AE were reported in the CQ and placebo group, respectively. The highest reported AEs for the CQ group occurred in the CNS, with about 18.7% of overall CQ participants reporting headache, dizziness, neuropathy, or other CNS-related AEs. In contrast, placebo group had higher records for respiratory distress, such as coughing, sore throat, or running nose.

The 18 HCQ studies contained 1,345 participants for HCQ-treated group and 1,330 participants for control group. Of these participants, 802 HCQ-treated participants and 807 control participants were part of the COVID-19 studies, while 543 HCQ-treated participants and 523 control participants were part of the non–COVID-19 studies. Total AEs reported for HCQ was 489 (36.4%), while total AEs reported for control was 228 (17.1%). GI AEs, such as diarrhea, nausea, liver damage, abdominal pain, and other non-specific GI AEs seemed to be the most dominant for both groups. Interestingly, cardiovascular AEs were reported in three of the studies (hypertension, acute coronary syndrome, and bradycardia) in non–COVID-19 patients that we examined. For COVID-19 studies, QT Prolongation was reported most frequently. Together, these stratified data provide ample information regarding the percentage of participants who experienced specific AEs.

### Subgroup Meta-Analysis for CQ and HCQ With Respect to Age, Duration, Dosage, and Treated Disorder

**Figure 6:**
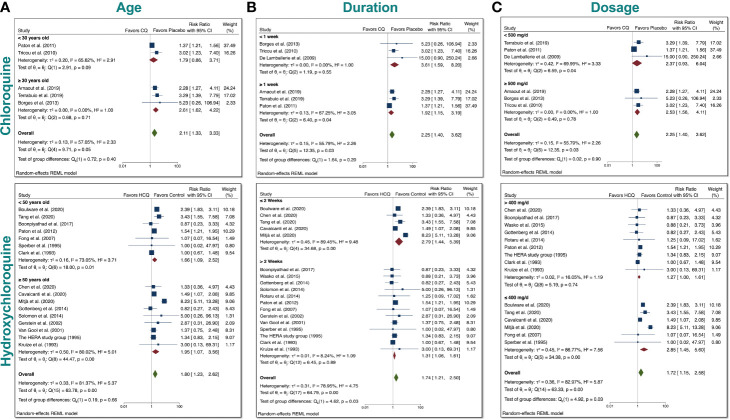
Subgroup meta-analyses for CQ and HCQ with respect to age, duration, dosage. We stratified the dosages used in the studies for both CQ and HCQ into two subgroups. We then performed subgroup analysis for dosage and trial duration. **(A)** For age, we separated CQ trials into <30 years old and ≥30 years old, while we separated HCQ trials into <50 years old and ≥50 years old. **(B)** For drug duration, we divided CQ studies into <1 week and ≥1 week, while we divided HCQ studies into <6 months and ≥6 months. **(C)** And for dosage, we wanted to investigate if there was a difference in using <500 mg/day versus using ≥500 mg/day for CQ, and ≥400 mg/day versus <400 mg/day for HCQ. Statistical data are presented in the figures.

Since we found a significant increase in total AEs when taking either drugs, we tested whether differences in age, duration, or dosage had any bearing on the results. We therefore performed subgroup meta-analysis. First, we examined age (**Figure 6A**). We divided the CQ trials into two groups: participants <30 years old and participants ≥30 years old. We stratified the HCQ trials into two groups: participants <50 years old and participants ≥50 years old. These ages were chosen to ensure that there was robust comparison, since the number of RCTs was very limited. We found that there was no group difference in either case, which suggests that age (younger vs. older) had no bearing on the total AEs experienced in participants.

Next, we assessed whether duration had any relevance to total AEs (**Figure 6B****)**. CQ trials were divided into two groups: <1 week and ≥1 week. Although there was no significant difference between the two groups for CQ, there was evidence of heterogeneity (I^2^ = 55.79%) between the two groups. It is important to note that when these studies were separately analyzed, there was statistical significance for either group (p < 0.05). Upon close inspection of the HCQ trials, we noted that trials for non–COVID-19 patients generally had longer duration than trials for COVID-19 patients. We therefore divided HCQ trials into two groups: ≤2 weeks and >2 weeks. This division allowed us to test whether there is a difference between RR with respect to trial duration for COVID-19 patients (shorter duration) and non–COVID-19 patients (longer duration). We found that with this division, there was a significant difference between the two test groups (p = 0.03), with evidence of overall heterogeneity between the two groups (I^2^ = 78.95%).

Furthermore, to determine if there were significant differences between a low versus a high dosage with respect to total AEs for either drug according to their respective median values. We stratified the dosages of the CQ studies into two groups: <500 mg/day and ≥500 mg/day (**Figure 6C**). This arbitrary grouping ensured that we included enough studies in each group for CQ, since the number of RCT for CQ is limited. There was no statistical group difference for CQ reports. For the HCQ studies, we used >400 mg/day and ≤400 mg/day, since this grouping divided the non–COVID-19 studies from the COVID-19 studies. As evident in our meta-analyses, there was significant difference between 2 subgroups for HCQ, in which the overall RR of total AEs was 1.72 (CI 95% 1.15–2.58, p < 0.05). Additionally, there was evidence of heterogeneity (I^2^ = 82.97%) between the two groups. Taken together, this indicates that a high dosage of HCQ (>400 mg/day) could lead to a significant increase in total AEs compared to a lower dosage.

Finally, we stratified for indication of use in another subgroup analysis to assess whether the treated disorders impacted total AEs (**Supplementary Figure S1**). The overall RR was 1.74 (CI 95% 1.21–2.50, p = 0.12), which indicates that the underlying pathologies did not significantly impact total AEs in HCQ-treated patients. Upon closer inspection, the overall RR of total AEs was significant in COVID-19 patients taking HCQ; however, the other non–COVID-19 conditions did not exhibit this trend. The subgroup analysis was not conducted in CQ group due to the limited number of studies.

Taken together, there was no statistical evidence to suggest that age (younger vs. older) differentially affected the total AEs when using either drug. In contrast, there was statistical evidence to suggest that dosage and duration has a significant impact on total AEs in the HCQ-treated patients.

### Meta-Regression Analyses for CQ and HCQ

Meta-regression analyses were performed to determine the relationship between RR and age, duration of trial, and dosage, as depicted in **Supplementary Figures S2, S3**. We examined if age of participants, duration of trial, or dosage has any effects on total AEs or withdrawals due to AEs. The size of the symbols indicates more weight toward a particular study. In all plots, the predicted regression lines and 95% confidence-interval lines are displayed. Regression of logarithm of RR of total AE with CQ and dosage revealed that dosage had an effect on total AEs. Age and duration of trial did not affect the total AEs for CQ.

## Discussion

The current pandemic with SARS-CoV-2 has relentlessly claimed thousands of lives and caused significant economic hardship. The urgent need for viable therapeutic options while vaccine development is in progress has resulted in the proposal of numerous antiviral medications (Beck et al., 2020). CQ and its derivative HCQ have been proposed as potential drugs to treat COVID-19. However, little is known regarding their safety profiles due to the lack of RCTs. To address this urgent issue, we performed a systematic review and meta-analysis by pooling the existing published data of AEs for CQ and HCQ relative to control.

It is important to note that CQ/HCQ used for the treatment of chronic diseases generally had a longer duration regimen and lower dosage (**Table 2**). To take this into account, we separated the COVID-19 studies from the non–COVID-19 ones. We found that the usage of either drug increased the relative risk (RR) for mild and total AEs in non–COVID-19 patients (**Figure 2**). Further system analyses showed that overall participants in the CQ trials experienced more neurologic, GI, dermatologic, and sensory AEs (**Figure 3**). However, we did not observe a significant elevation in any of these AEs in HCQ-treated non–COVID-19 patients relative to control patients.

COVID-19 studies included five trials from patients treated with HCQ. We found a significant increase in mild and total AEs in HCQ-treated COVID-19 patients relative to control patients (**Figure 4**). Dermatologic, GI, sensory, and cardiovascular AEs were significantly elevated in COVID-19 patients treated with HCQ. Although cardiovascular AEs was not as common in the non–COVID-19 patients, it was more prevalent in the COVID-19 patients. This may be due to an increase in dosage given to COVID-19 patients.

Given the severity of cardiovascular AEs, it is critical to note that six studies reported cardiovascular AEs including hypertension, acute coronary syndrome, bradycardia, and QT prolongation (Gottenberg et al., 2014; Rotaru ea, 2014; Cavalcanti et al., 2020; Mitja et al., 2020; Tang et al., 2020). Although there were no cardiovascular AEs reported in the CQ studies that we analyzed, its cardiotoxicity has also been noted in a plethora of studies (Chatre et al., 2018). An excellent systematic review article by Chatre et al. documented cardiac complications that are attributed to CQ and HCQ (Chatre et al., 2018). In their review, they found that among other cardiovascular complications, conduction bundle or atrioventricular block were reported more frequently. Moreover, QT interval prolongation has been noted in numerous studies (Rey et al., 2003; Morgan et al., 2013; Chorin et al., 2020; van den Broek et al., 2020) and has also been found in studies involving COVID-19 patients (Cavalcanti et al., 2020). Severely prolonged QT interval can lead to lethal arrhythmias and sudden cardiac death. Therefore, the prevalence of these cardiovascular AEs warrants periodic electrocardiogram (ECG) monitoring when participants are undergoing these therapies, as cardiovascular AEs can be fatal.

Overall, participants who took CQ exhibited more AEs (40.4%) relative to control (25.5%, **Figure 5**). In the HCQ studies, 36.4% of total AEs were reported versus 17.1% for control. The high percentage of total AEs occurring with CQ participants is concerning, but consistent with the consensus that HCQ is a safer alternative to CQ (McChesney, 1983; Finbloom et al., 1985; Felson et al., 1990; Liu et al., 2020). When total AEs were stratified according to different organ systems, we found that CQ had more participants exhibiting CNS AEs (18.7%), while HCQ participants had more participants experiencing GI AEs (31.5%). It is worth noting that only 10.4% of HCQ participants exhibited CNS AEs. The extra hydroxyl group in HCQ may decrease the occurrence of CNS AEs. More mechanistic, controlled studies need to be performed to confirm this finding.

Furthermore, subgroup analyses (**Figure 6**) of CQ reports revealed no evidence in differences of RR of total AEs when studies were divided by age (younger vs. older), dosage (lower vs. higher) and duration (shorter vs. longer). When we performed meta-regression analyses (**Supplementary Figure S2**), there was a relationship between dosage and total AEs in the CQ group, which suggests that the subgroup meta-analyses for dosage would be more robust if more CQ RCTs existed. In contrast, subgroup analysis of HCQ reports suggested that lower duration (<2 weeks, **Figure 6B**) and higher dosage of HCQ (≥400 mg/day) could lead to more total AEs (**Figure 6C**). Indeed, the duration and dosage regimen of HCQ significantly differ for COVID-19 patients and non–COVID-19 patients. COVID-19 patients received higher dosage for a shorter duration, while non–COVID-19 patients received a lower dosage for a longer duration.

Given the long half-life of HCQ (Tett et al., 1989), it is plausible that the longer the duration of dosing regimen, or the higher the dosage, the more total AEs would be observed. Therefore, caution is recommended when taking higher dosage or longer duration of HCQ. Although we did not find a difference in total AEs when accounting for the different treated disorders (**Supplementary Figure S1**), this may be due to the limited number of studies for each disorder. However, upon closer inspection, there is evidence that COVID-19 patients experienced an overall RR of total AEs that was in favor of the control, while non–COVID-19 treated conditions such as rheumatoid arthritis and diabetes did not. Therefore, it is important to consider the underlying condition when examining the presented data, as this affects the dosing schedule and duration, which consequently impacts the occurrence and type of AEs.

### Limitations

Here, we present a comprehensive analysis that reveals the increase in AEs associated with either CQ or HCQ. However, RCTs have several limitations when it comes to identifying adverse drug reactions or adverse events, including under-reporting, poor reporting, and lack of information on long-term outcomes. In addition, this systematic review and meta-analysis is limited due to the lack of large RCTs. For instance, although we did not observe an increase in severe AEs associated with taking either medication, there has been numerous records showing cardiovascular AEs. Moreover, due to the sparse RCTs, the analyses reported may be affected in a few instances according to the sensitivity analyses performed. These analyses took into account removing one study (**Supplementary Figures S8–S10**), or removal of all the studies that did not report any events (**Supplementary Figures S11–S13**). In this study, by including all the known RCTs in the meta-analysis, we were able to more confidently report our findings. Despite including all these studies, however, this meta-analysis would benefit significantly from larger RCTs, as this would provide better representations of both drugs’ safety profiles. Indeed, several RCTs are currently ongoing that involve both medications, which would help drive future analyses.

## Conclusions

Taken together, our data show that participants taking either CQ or HCQ experienced more mild and total AEs relative to placebo control. Precautionary measures should be taken when giving these medications for their therapeutic impact.

## Author Contributions

LR and PT designed the study. LR and PT screened and evaluated studies. LR performed statistical analyses. SY checked studies included. PT and SY checked statistical analyses. LR, WX, JO, and PT performed comprehensive characterization of studies. SY and NC provided expertise. LR, PT, and NC wrote the manuscript. All authors contributed to the article and approved the submitted version.

## Funding

This work was supported by American Heart Association Predoctoral Award 18PRE34030199 (LR); Postdoctoral Fellowship from NIH F32 HL149288, Postdoctoral Fellowship from NIH T32 HL086350 Training Program in Basic and Translational Cardiovascular Science (PT); NIH R01 HL085727, HL085844, and HL137228, Research Award from the Rosenfeld Foundation, VA Merit Review Grant I01 BX000576 and I01 CX001490 (NC). The contents of this article do not represent the views of the funding agencies. This manuscript has been released as a pre-print at medRxiv, Ren et al., (2020). NC is the holder of the Roger Tatarian Endowed Professorship in Cardiovascular Medicine and a part-time staff physician at VA Northern California Health Care System, Mather, CA, USA.

## Conflict of Interest

The authors declare that the research was conducted in the absence of any commercial or financial relationships that could be construed as a potential conflict of interest.

## Conflict of Interest

The authors declare that the research was conducted in the absence of any commercial or financial relationships that could be construed as a potential conflict of interest.
