# Supplementary material for: Assessment of Chloroquine and Hydroxychloroquine Safety Profiles: A Systematic Review and Meta-Analysis
Source: Front Pharmacol. 2020 Oct 14;11:562777. doi: 10.3389/fphar.2020.562777 (PMC7591721; doi:10.3389/fphar.2020.562777)
Supplement: Supplementary file 2 [file DataSheet_2.pdf]

Assessment of Chloroquine and Hydroxychloroquine Safety Profiles: A Systematic Review and Meta-Analysis

Lu Ren[1], MD, Wilson Xu[1], BS, James L Overton[1], MS, Shandong Yu[3], MD, PhD, Nipavan Chiamvimonvat[1,2,*], MD, and Phung N. Thai[1,*], PhD

[1]Department of Internal Medicine, Cardiology, UC Davis; [2]Department of Veteran Affairs; [3]Department of Cardiology, Cardiovascular Center, Beijing Friendship Hospital, Capital Medical University, Beijing

Corresponding authors:

Phung N. Thai and Nipavan Chiamvimonvat
Division of Cardiovascular Medicine
University of California, Davis
451 Health Science Drive, GBSF 6315
Davis, CA 95616
Department of Veterans Affairs, Northern California Health Care System
10535 Hospital Way
Mather, CA 95655
Emails: pnthai@ucdavis.edu; nchiamvimonvat@ucdavis.edu

Supplementary Figures

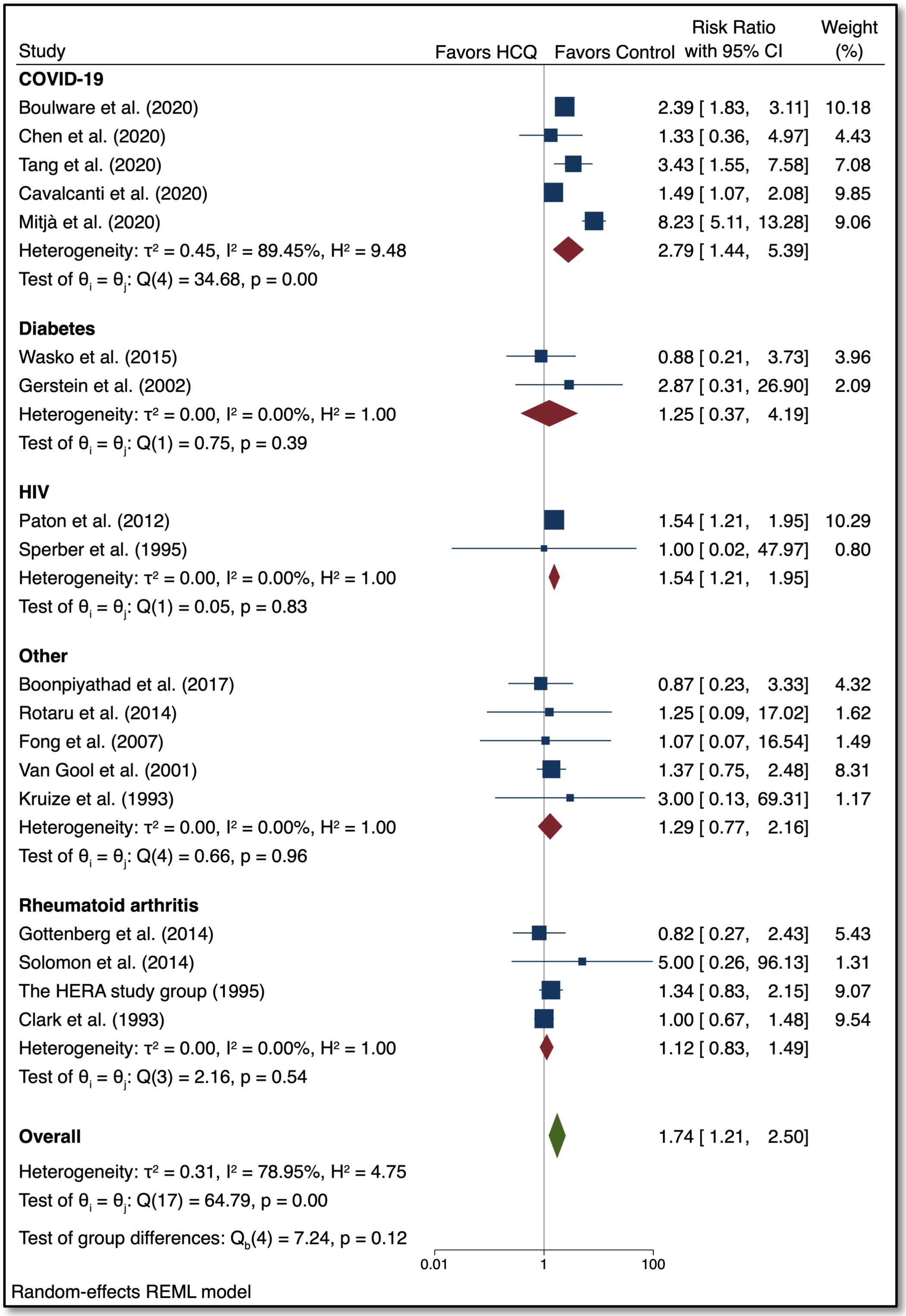

Supplementary Figure S1. Subgroup analysis of treated disorders for HCQ group.

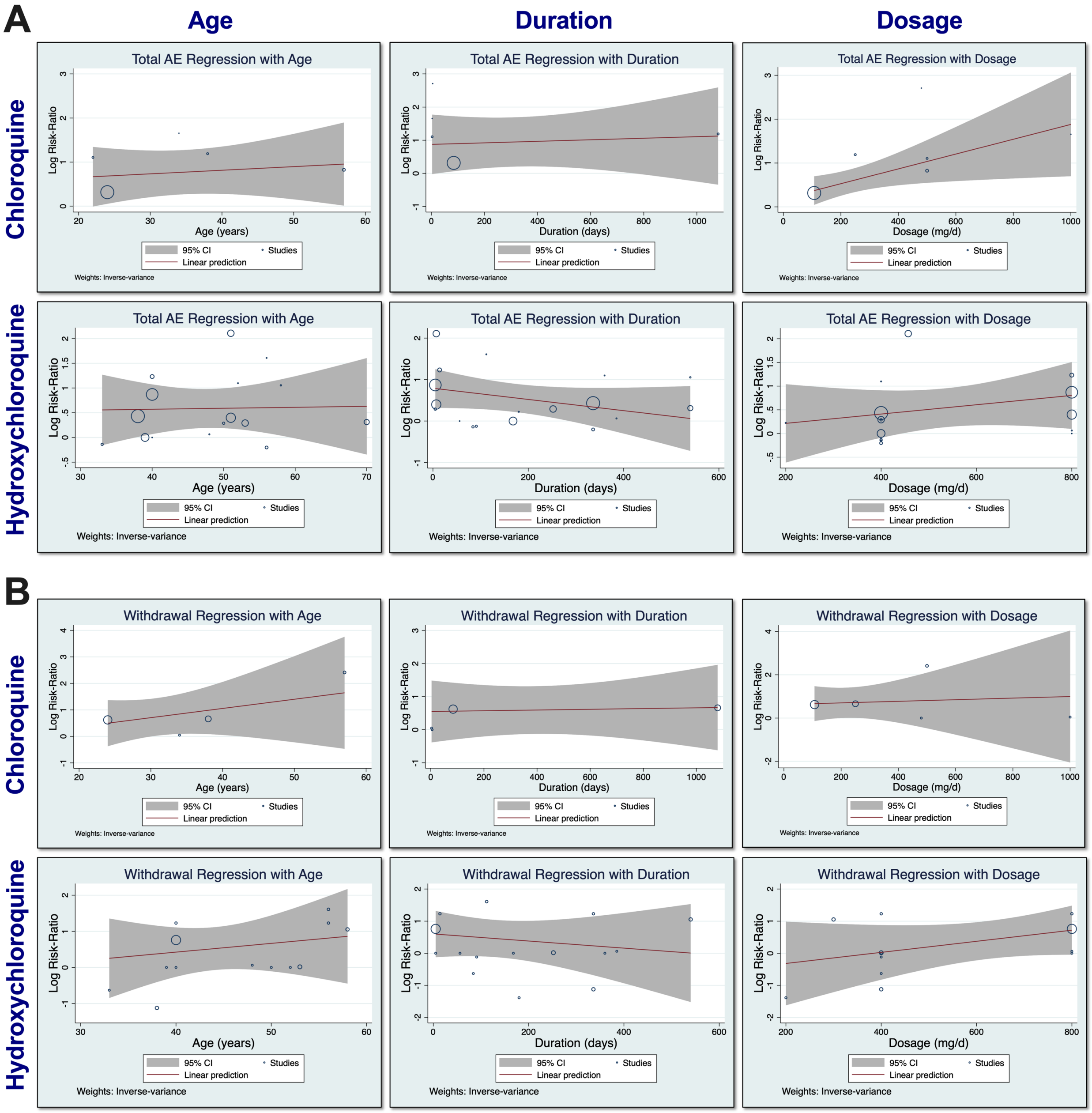

Supplementary Figure S2. Meta-regression analyses for CQ and HCQ. Meta-regression analyses were performed using the log RR of total AEs with respect to age, duration, and dosage for both CQ and HCQ, as shown in panel **A**). Panel **B)** depicts the meta-regression analyses of RR of withdrawals due to AEs with respect to age, duration, and dosage for both CQ and HCQ. Statistical data are presented in the figure.

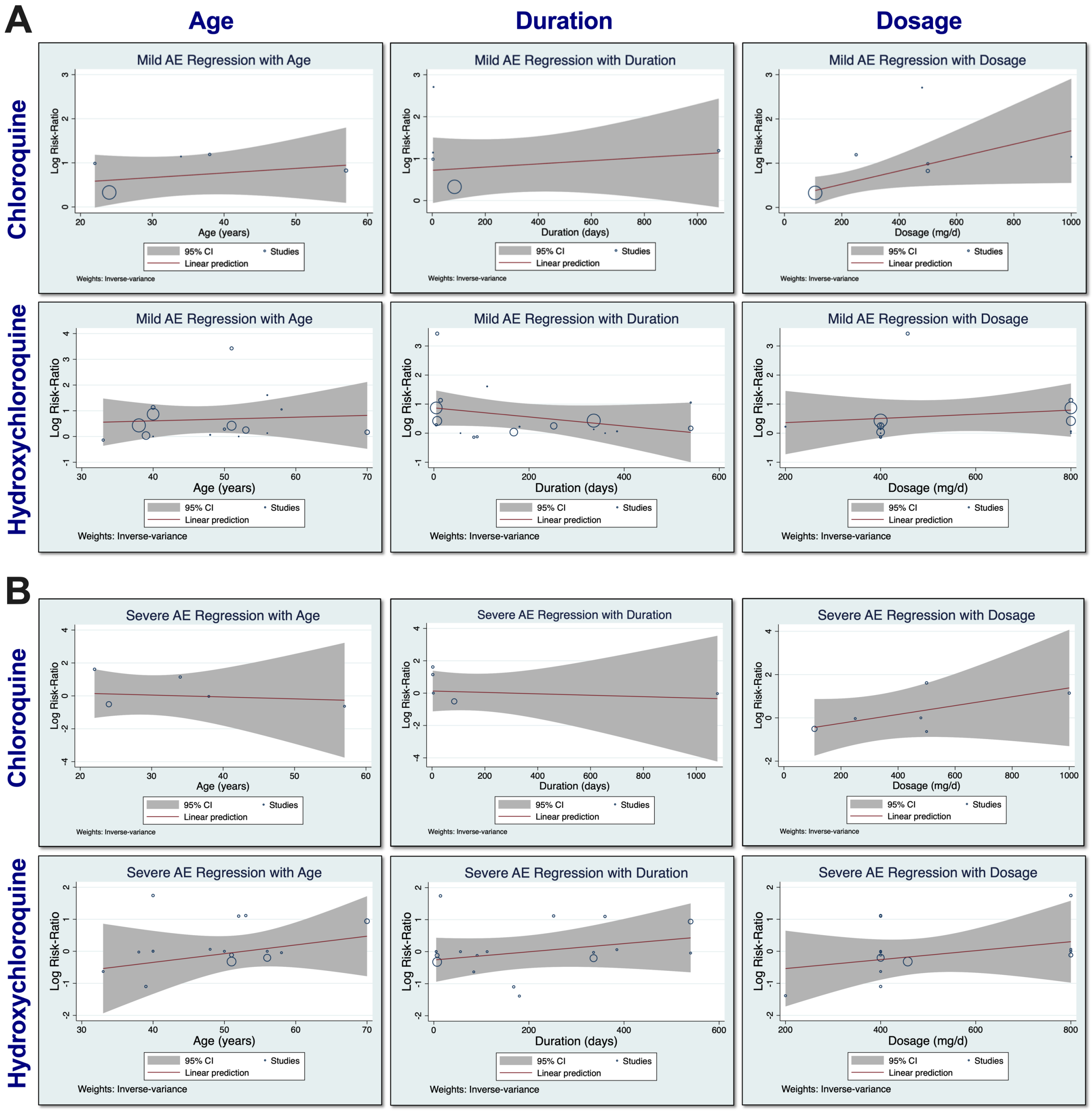

Supplementary Figure S3. A) Meta-regression analyses of mild and **B**) severe AEs with respect to age, duration, and dosage.

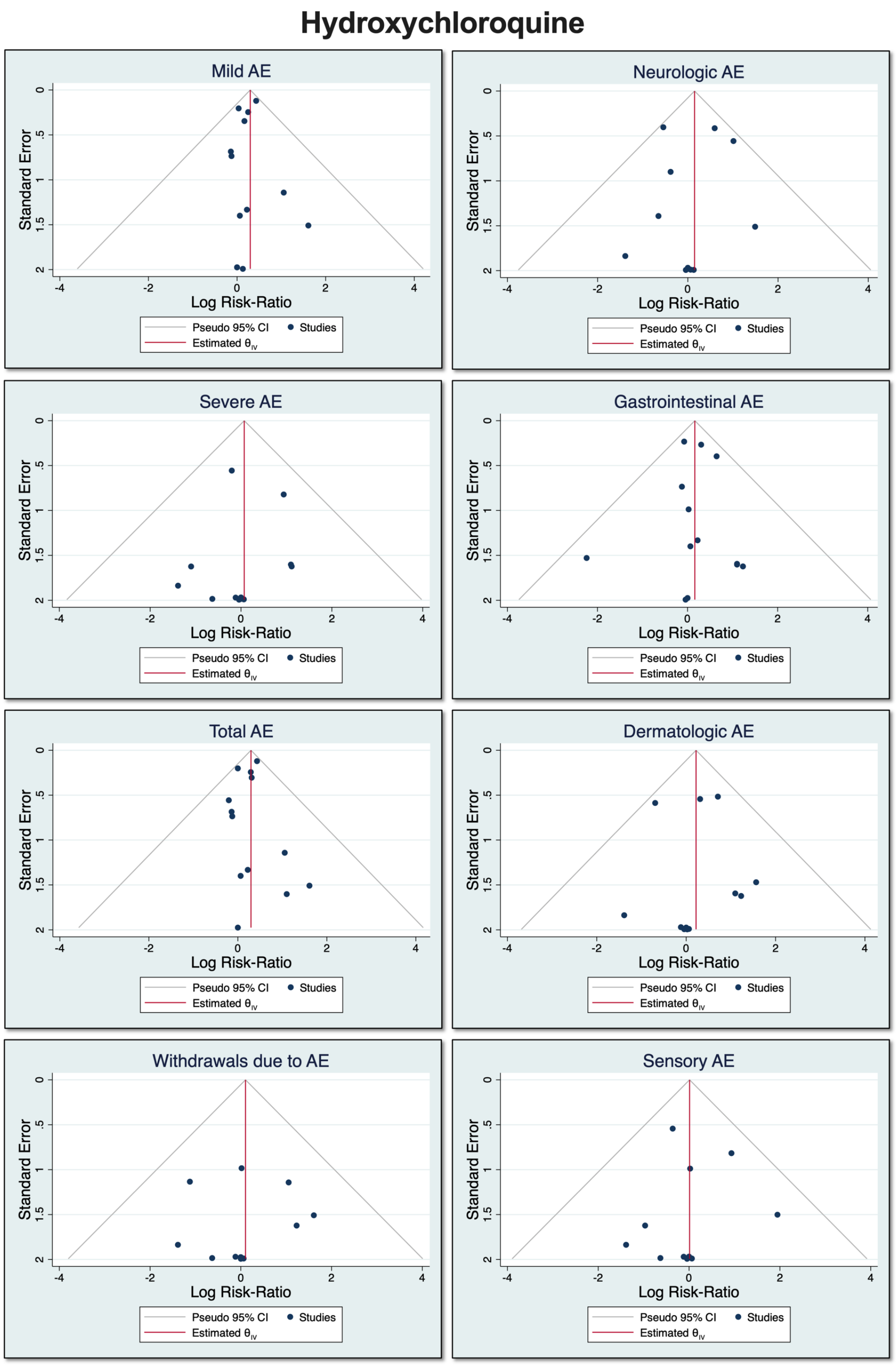

Supplementary Figure S4. Funnel plots showing publication bias for mild, severe, total AEs, withdrawals due to AEs, neurologic, gastrointestinal, dermatologic, and sensory AEs.

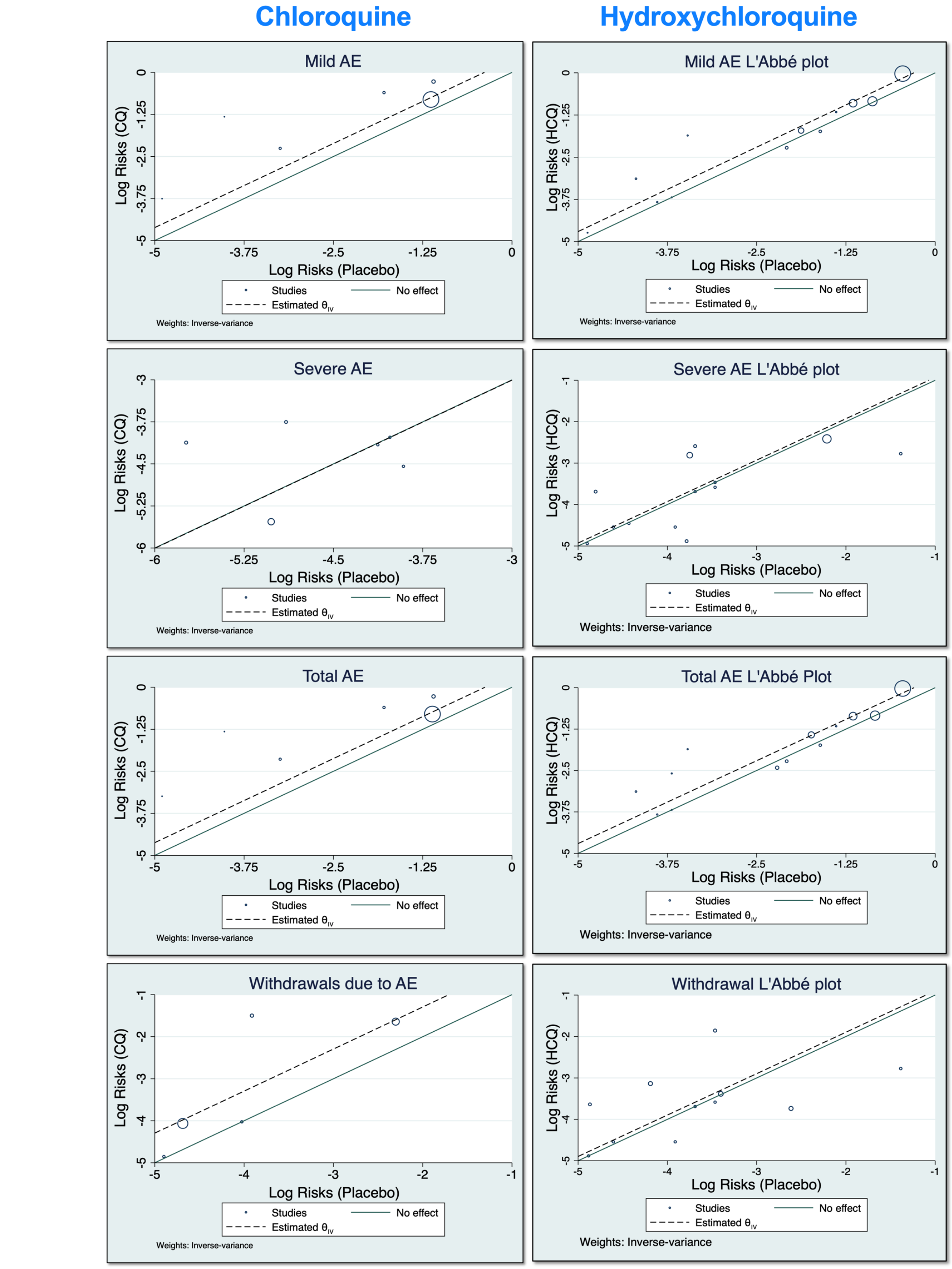

Supplementary Figure S5. L'Abbé Plots examining heterogeneity for mild AEs, severe AEs, total AEs, and withdrawals due to AEs.

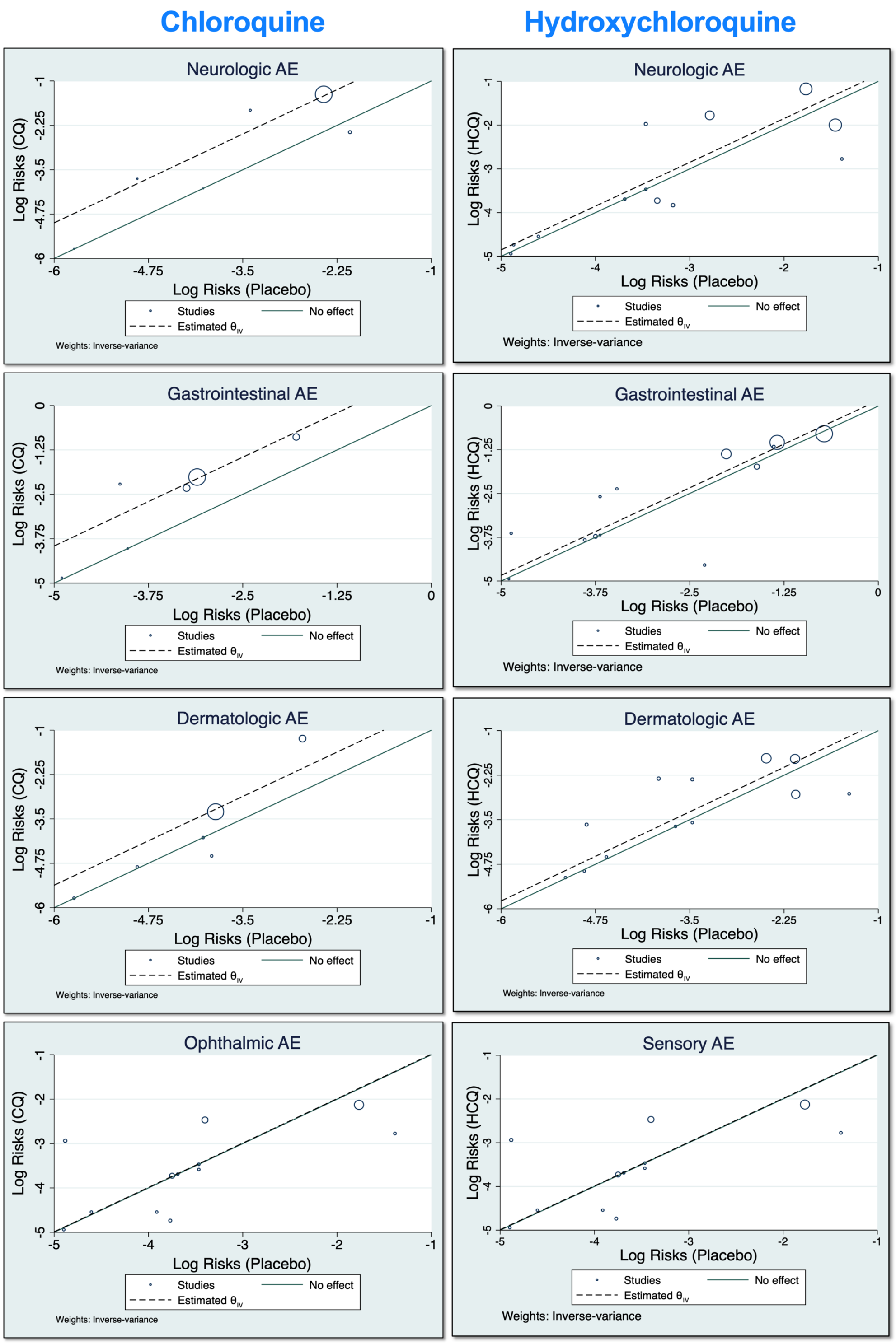

Supplementary Figure S6. L'Abbé Plots examining heterogeneity for neurologic, gastrointestinal, dermatologic, and ophthalmic/sensory AEs.

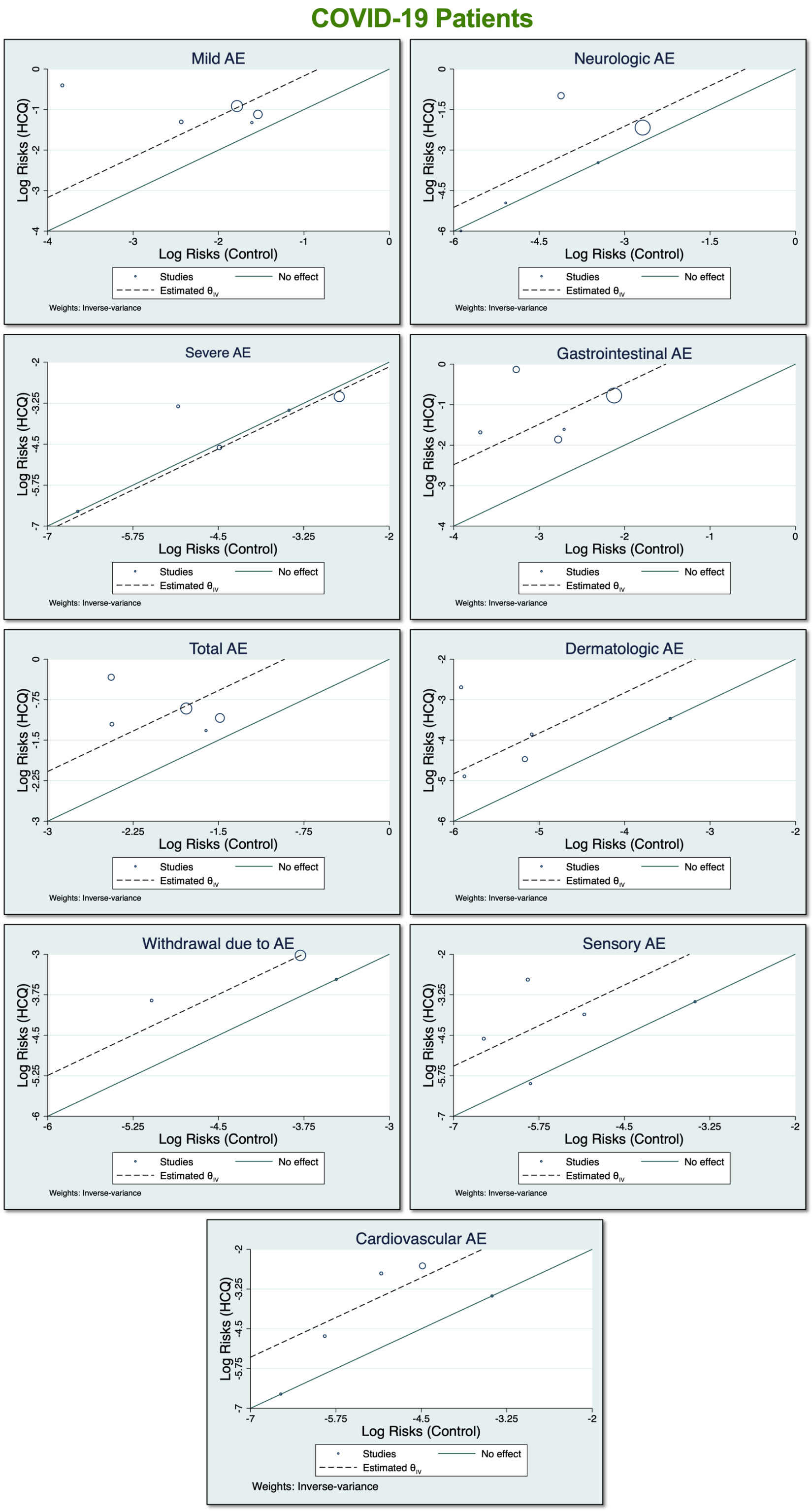

Supplementary Figure S7. L'Abbé Plots examining heterogeneity for mild, severe, total, withdrawal, neurologic, gastrointestinal, dermatologic, sensory, and cardiovascular AEs in COVID-19 studies using hydroxychloroquine.

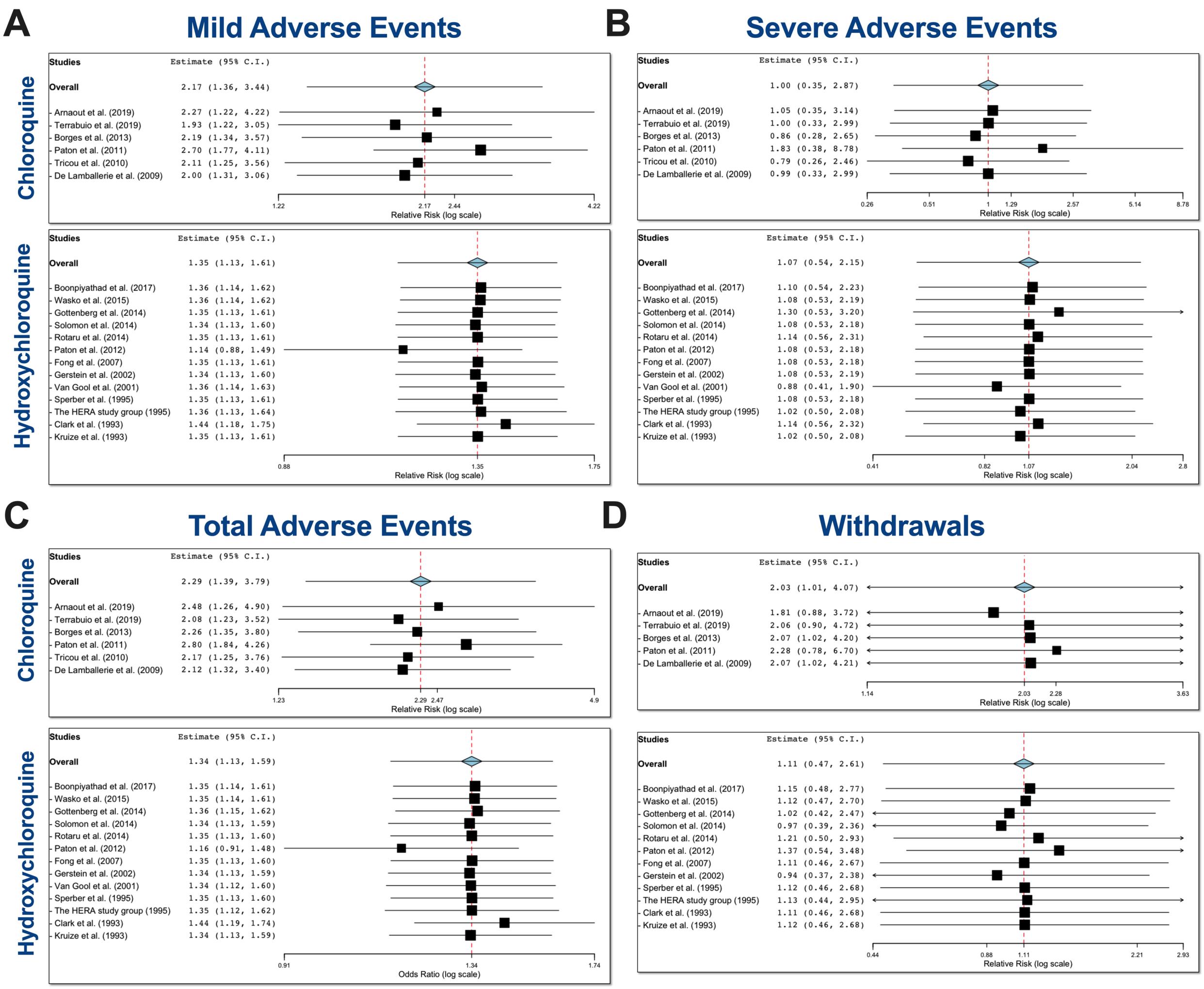

Supplementary Figure S8. Sensitivity analysis for mild, severe, total, withdrawal AEs in non-COVID-19 studies. Forest plots show the new 95% CI when a particular study is removed.

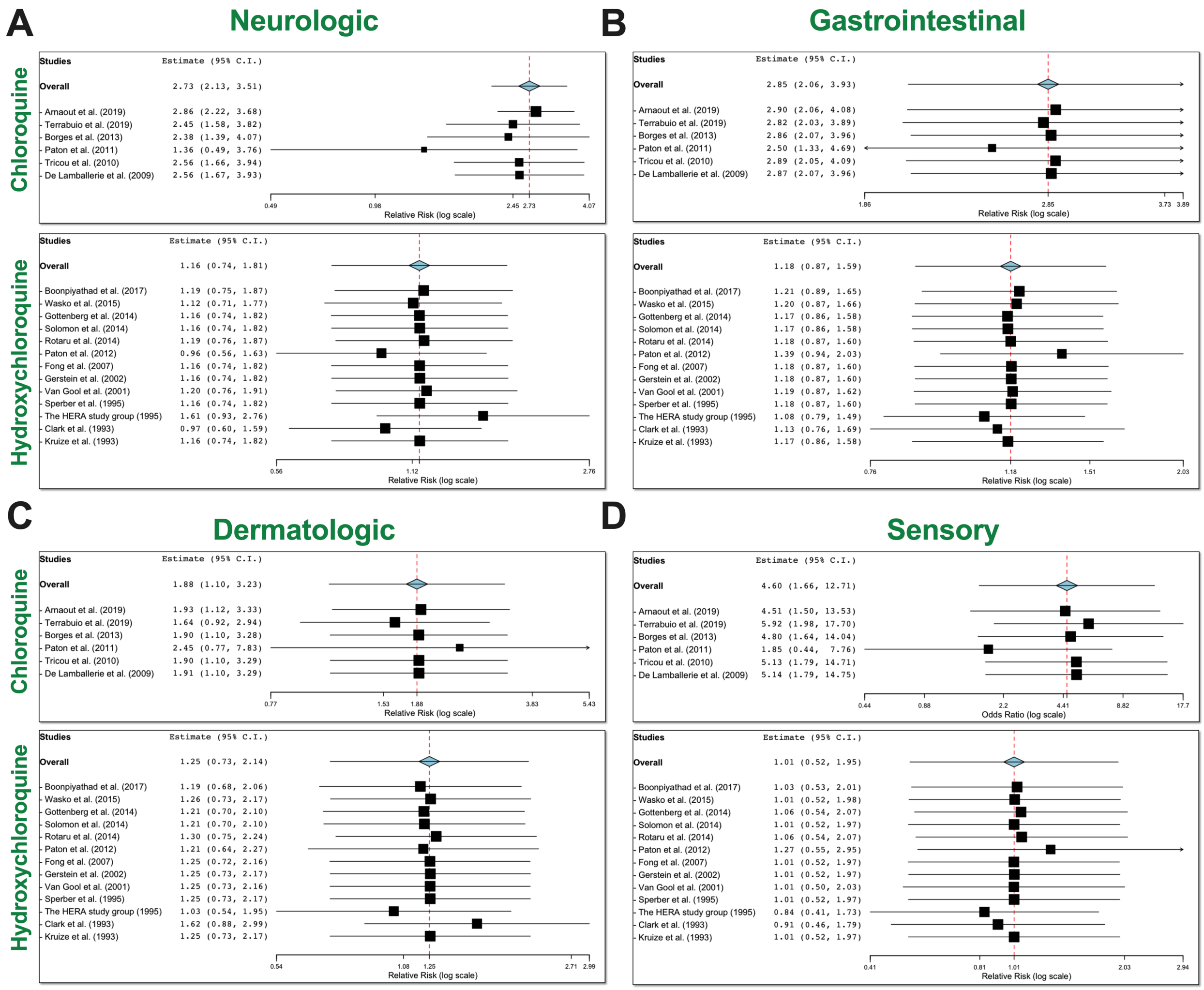

Supplementary Figure S8. Sensitivity analysis for neurologic, gastrointestinal, dermatologic, and sensory AEs in non-COVID-19 studies. Forest plots show the new 95% CI when a particular study is removed.

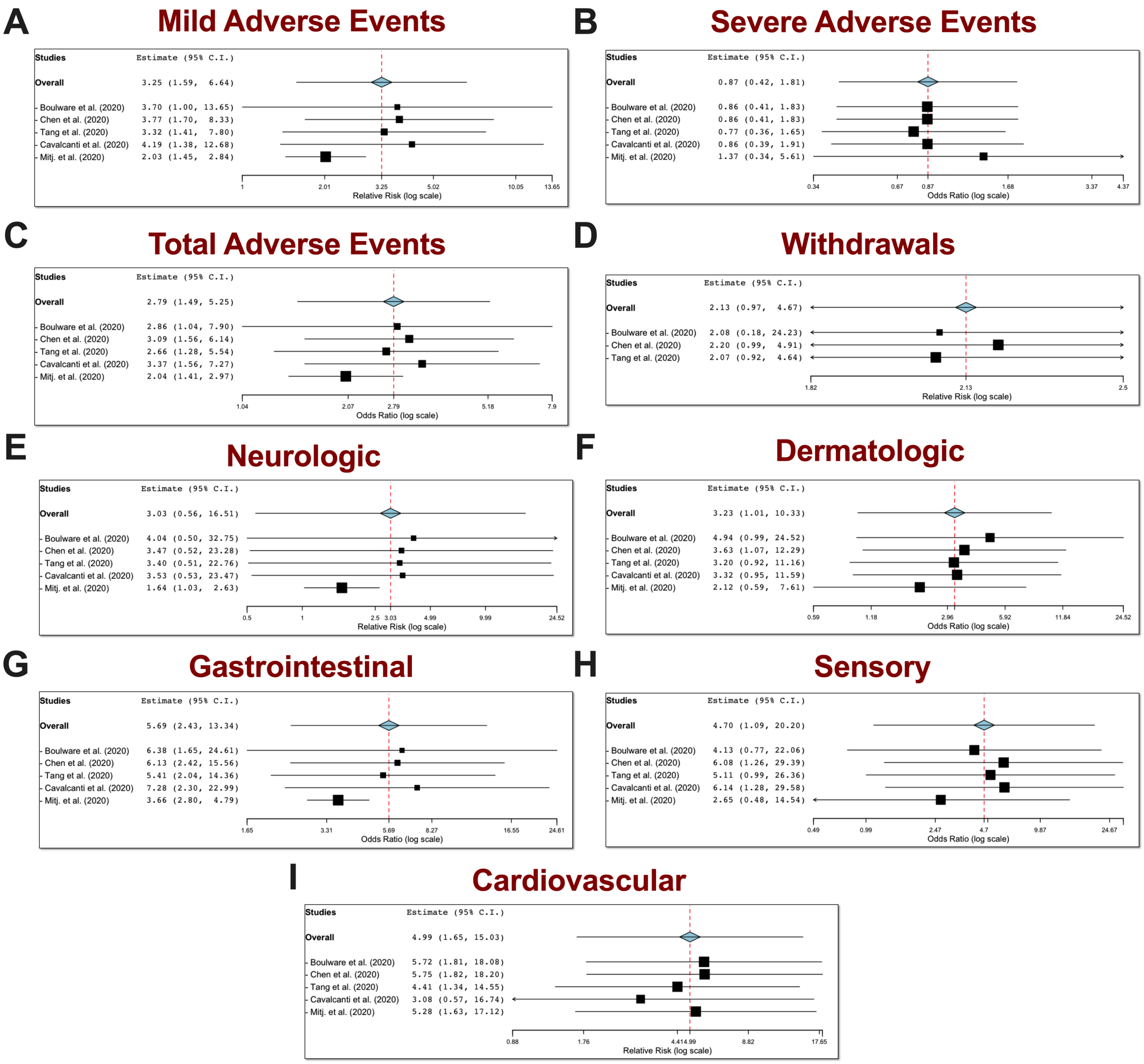

Supplementary Figure S10. Sensitivity analysis for mild, severe, total, withdrawals, neurologic, dermatologic, gastrointestinal, sensory, and cardiovascular AEs in COVID-19 studies. Forest plots show the new 95% CI when a particular study is removed.

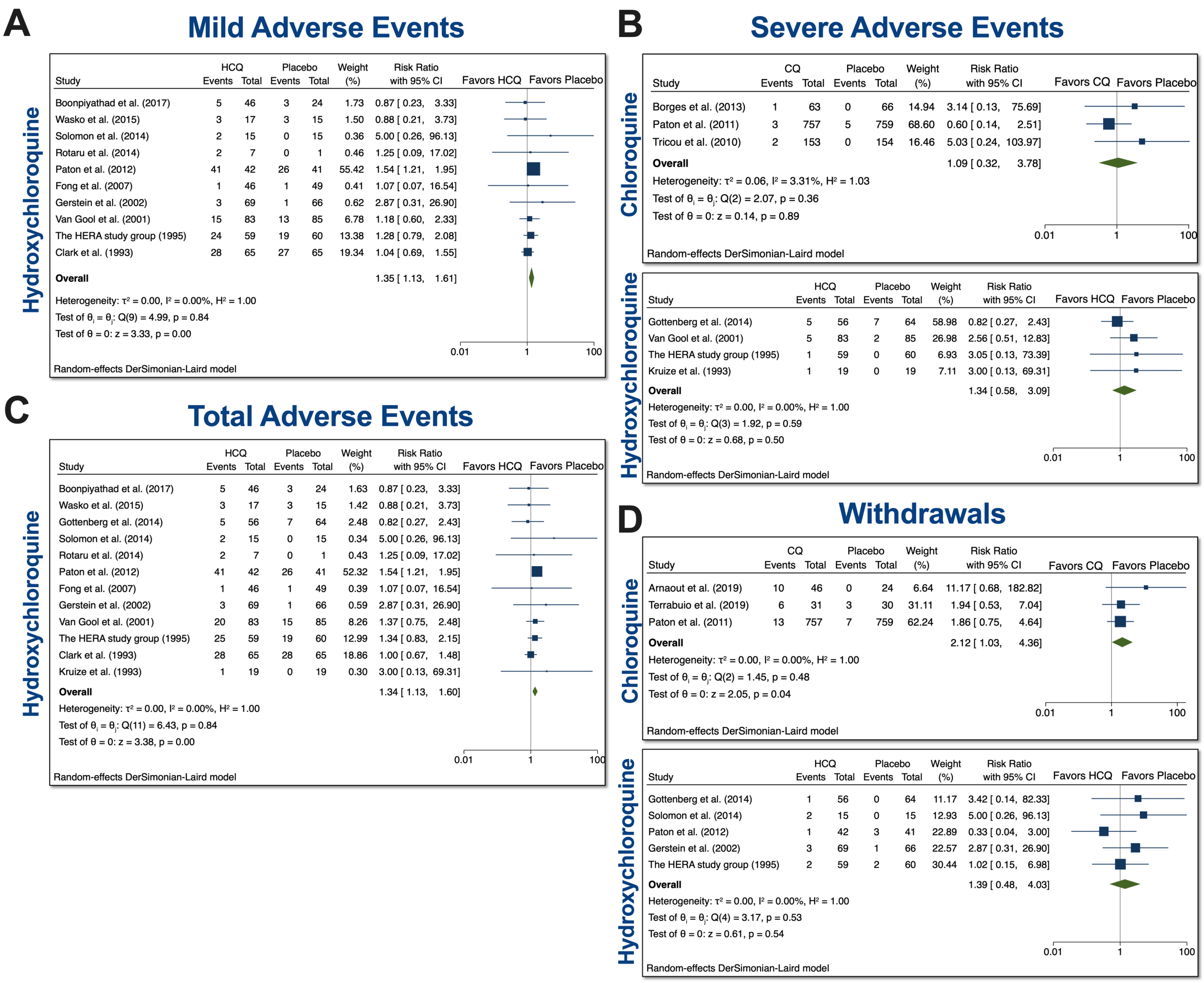

Supplementary Figure S10. Sensitivity analysis for mild, severe, total, and withdrawals in non-COVID-19 studies. Forest plots show the new 95% CI when the zero event studies were removed.

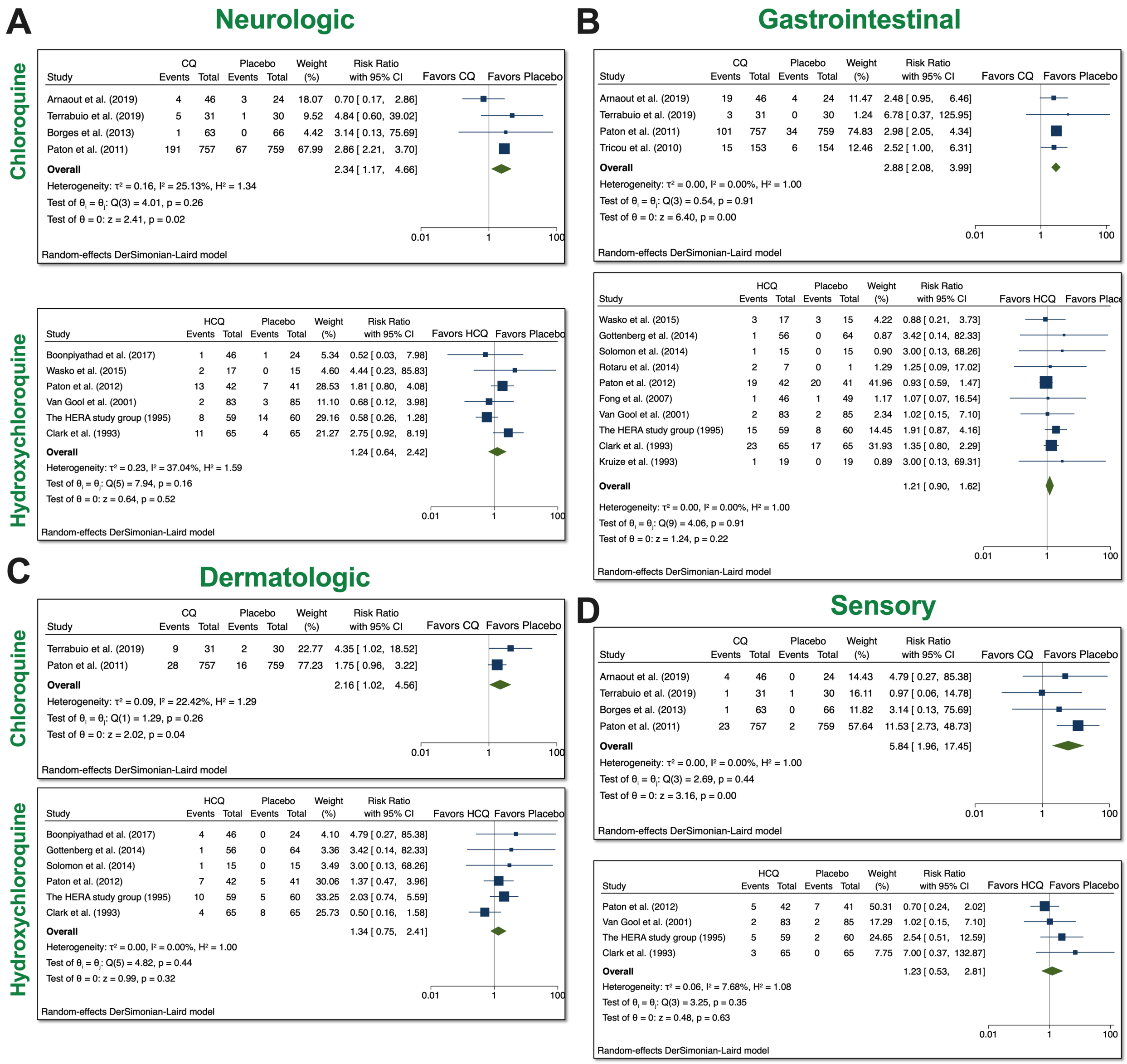

Supplementary Figure S10. Sensitivity analysis for neurologic, gastrointestinal, dermatologic, and sensory in non-COVID-19 studies. Forest plots show the new 95% CI when the zero event studies were removed.

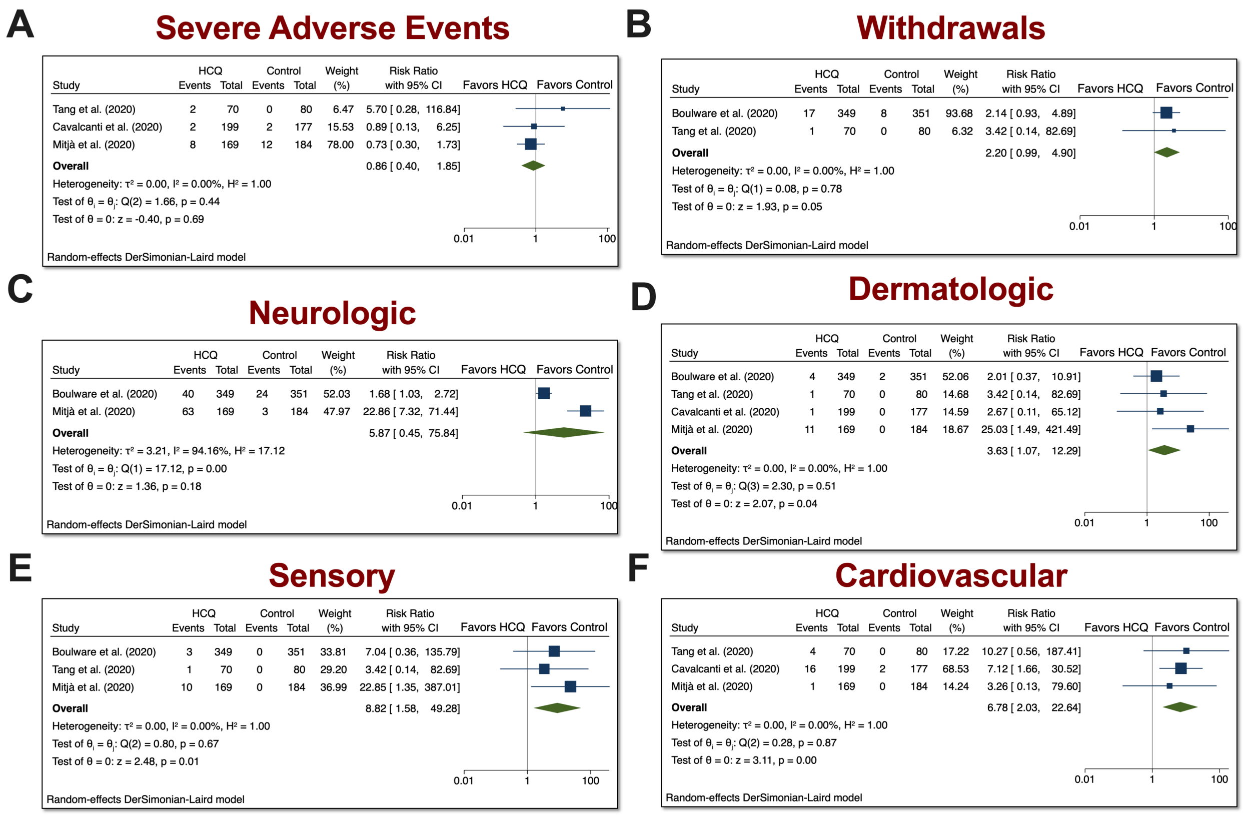

Supplementary Figure S11. Sensitivity analysis for severe, withdrawals, neurologic, dermatologic, sensory, and cardiovascular AEs in COVID-19 studies. Forest plots show the new 95% CI when the zero event studies were removed.

Supplementary Table S1. Risk of Bias Assessment

Chloroquine Studies

Study	Random Sequence Generation	Allocation Concealment	Selective Reporting	Other Sources of Bias	Blinding (Participants and Personnel)	Blinding (Outcome Assessment)	Were adverse events actively sought	Were adverse events categorized using standard classifications
*Arnaout et al. (2019)	+	-	+	+	+	-	+	+
*Terrabuio et al. (2018)	+	+	+	-	+	+	+	+
*Borges et al. (2013)	-	-	+	+	+	-	+	-
*Paton et al. (2011)	+	+	+	+	+	+	+	+
*Tricou et al. (2010)	+	+	+	+	+	+	+	+
*De Lamballerie et al. (2009)	+	-	+	+	+	-	+	-

Hydroxychloroquine Studies

Study	Random Sequence Generation	Allocation Concealment	Selective Reporting	Other Sources of Bias	Blinding (Participants and Personnel)	Blinding (Outcome Assessment)	Were adverse events actively sought	Were adverse events categorized using standard classifications
*Chen et al. (2020)	+	+	+	+	+	+	+	+
*Boonpiyathad et al. (2017)	+	+	+	+	-	-	-	-
*Wasko et al. (2016)	+	+	+	+	+	+	+	+
*Gottenberg et al. (2014)	+	+	+	+	+	+	+	+
*Solomon et al. (2014)	+	+	+	+	+	+	+	-
*Rotaru et al. (2014)	+	+	+	+	+	-	+	-
*Paton et al. (2012)	+	+	+	+	+	+	+	+
*Fong et al. (2007)	+	+	+	+	+	+	+	-
*Gerstein et al. (2001)	+	+	+	+	+	+	+	+
*Van Gool et al. (2001)	+	+	+	+	+	+	+	+
*Sperber et al. (1995)	+	+	+	+	+	+		
*The HERA Study Group (1995)	+	+	+	+	+	+	+	+
*Clark et al. (1993)	+	+	+	+	+	+	+	+
*Kruize et al. (1993)	+	+	+	+	+	+	+	-
*Boulware et al. (2020)	+	+	+	+	+	+	+	+
*Tang et al. (2020)	+	+	+	+	-	+	+	+
Cavalcanti et al. (2020)	+	+	+	+	-	+	+	+
Mitjà et al. (2020)	+	+	+	+	-	-	+	+

Supplementary Table S1. Risk of Bias Assessment

Search Results

CQ total=2577

MEDLINE

chloroquine and "side effects": 651
chloroquine and "adverse events": 267 chloroquine and "toxicity": 73
chloroquine and “randomized controlled trial”: 778 chloroquine and "adverse effects": 335

=2104

Cochrane Central Register of Controlled Trials

Chloroquine and “randomized controlled trial”: 74 chloroquine and "side effects": 110
chloroquine and "adverse events": 140 chloroquine and "toxicity": 60

chloroquine and "adverse effects": 62 =446

ClinicalTrials.gov

Chloroquine and clinical trial: 27

HCQ total=1689
MEDLINE
hydroxychloroquine and "side effects": 249 hydroxychloroquine and "adverse events": 151 hydroxychloroquine and "toxicity": 568 hydroxychloroquine and “randomized controlled trial”: 241 hydroxychloroquine and "adverse effects": 63
=1272

Cochrane Central Register of Controlled Trials

hydroxychloroquine and “randomized controlled trial”: 62 hydroxychloroquine and "side effects": 68 hydroxychloroquine and "adverse events": 128 hydroxychloroquine and "toxicity": 74 hydroxychloroquine and "adverse effects": 38

=370

ClinicalTrials.gov

hydroxychloroquine and clinical trial: 46 **Reference searching**: 1
